# Prognostic value of FLOT1-related gene signature in head and neck squamous cell carcinoma: insights into radioresistance mechanisms and clinical outcomes

**DOI:** 10.1038/s41420-025-02500-1

**Published:** 2025-05-07

**Authors:** Min Kyeong Lee, Seon Rang Woo, Joo Kyung Noh, MinJi Bae, YeonSeo Lee, Soonki Min, Moonkyoo Kong, Young Chan Lee, Seong‐Gyu Ko, Young-Gyu Eun

**Affiliations:** 1 Department of Biomedical Science and Technology, Graduate School, Kyung Hee University, Seoul, Republic of Korea; 2 Department of Otolaryngology-Head and Neck Surgery, Kyung Hee University School of Medicine, Kyung Hee University Medical Center, Seoul, Republic of Korea; 3 Department of Radiation Oncology, Kyung Hee University School of Medicine Kyung Hee University Medical Center, Seoul, Republic of Korea; 4 Department of Preventive Medicine, College of Korean Medicine, Kyung Hee University, Seoul, Republic of Korea

**Keywords:** Oral cancer detection, Apoptosis

## Abstract

We aimed to develop and validate the ability of a FLOT1-related gene signature to predict survival in head and neck squamous cell carcinoma (HNSCC) patients and to explore FLOT1’s role in modulating the responses to radiation therapy (RT). Using TCGA dataset, we identified a gene expression signature reflective of FLOT1 and applied LASSO regression to build a prediction model. Patients were stratified into high- and low-risk subgroups based on this signature. The prognostic value was confirmed across three independent cohorts, showing that high-risk patients had significantly poorer overall survival. Cox proportional hazards models were used to establish this gene signature as an independent prognostic factor for overall survival in HNSCC patients. Additionally, this signature predicted survival outcomes in patients undergoing RT. In vitro and in vivo experiments revealed that inhibiting FLOT1 expression increased the radiation sensitivity of HNSCC cells by modulating the phospho-PTEN/IGF1R axis. Moreover, silencing FLOT1 decreased radioresistance in radioresistant cell lines and xenograft mouse models. In conclusion, the FLOT1-related gene signature is a strong prognostic marker for HNSCC and may help identify patients who may benefit from RT.

## Introduction

Head and neck squamous cell carcinoma (HNSCC) occurs in the mucosal epithelia of the oral cavity, pharynx, hypopharynx, and larynx. It is one of the most common types of cancer with an annual incidence of over half a million cases [1]. HNSCC has a high recurrence rate and poor prognosis. Despite the advancements in technology and treatment, survival rates have not improved significantly in recent years. Overall, the 5-year survival rate of HNSCC patients is ~50% [2]. Around 50% of the patients are diagnosed with locally advanced cancer. Radiation therapy (RT) is a key part of treatment for these patients, but the response and prognosis depend on various clinical factors, including tumor stage, location, and human papilloma virus (HPV) status [3, 4]. Additionally, the response to RT and clinical outcomes varies among patients. Consequently, high rates of side effects are associated with RT for HNSCC [5, 6]. In recent decades, efforts have been made to identify subsets of patients who can benefit most from RT [7]. In this study, we attempted to offer evidence and insights into the use of RT response-related genes as potential signatures to identify patients with radiosensitive cancer or as targets to promote personalized radiation.

Flotillin-1 (FLOT1), a lipid raft protein, participates in various cellular functions, including cell adhesion, actin cytoskeleton reorganization, endocytosis, phagocytosis, signal transduction, and cell proliferation [8, 9]. FLOT1 has been reported in multiple types of cancers, including breast cancer, gastric cancer, hepatocellular carcinoma, and HNSCC [10–13]. In breast cancer, inhibiting FLOT1 expression in breast cancer has been reported to be involved in the proliferation and tumorigenicity of breast cancer cells in vitro and in vivo [14]. In gastric cancer, FLOT1 expression is associated with poor disease-free survival [15]. In hepatocellular carcinoma, FLOT1 overexpression enhances insulin signaling, which promotes proliferation and metastasis [16]. In HNSCC, the upregulation of FLOT1 induces proliferation and epithelial-mesenchymal transition (EMT) signaling. FLOT1 plays an important role in the occurrence, development, and prognosis of tumors [17]. However, despite increasing evidence supporting the involvement of FLOT1 in HNSCC progression, the clinical relevance of FLOT1 has yet to be properly examined. Furthermore, the effects of FLOT expression on RT in HNSCC and the exact mechanisms underlying radioresistance remain unclear.

In this study, we systematically characterized genomic data from The Cancer Genome Atlas (TCGA) cohorts of HNSCC patients to develop molecular signatures based on FLOT1-related gene expression patterns. Additionally, we aimed to elucidate the mechanisms underlying radioresistance in HNSCC to identify potential therapeutic strategies.

## Results

### Identification of FLOT1-related gene signature in training cohort

**Fig. 1:**
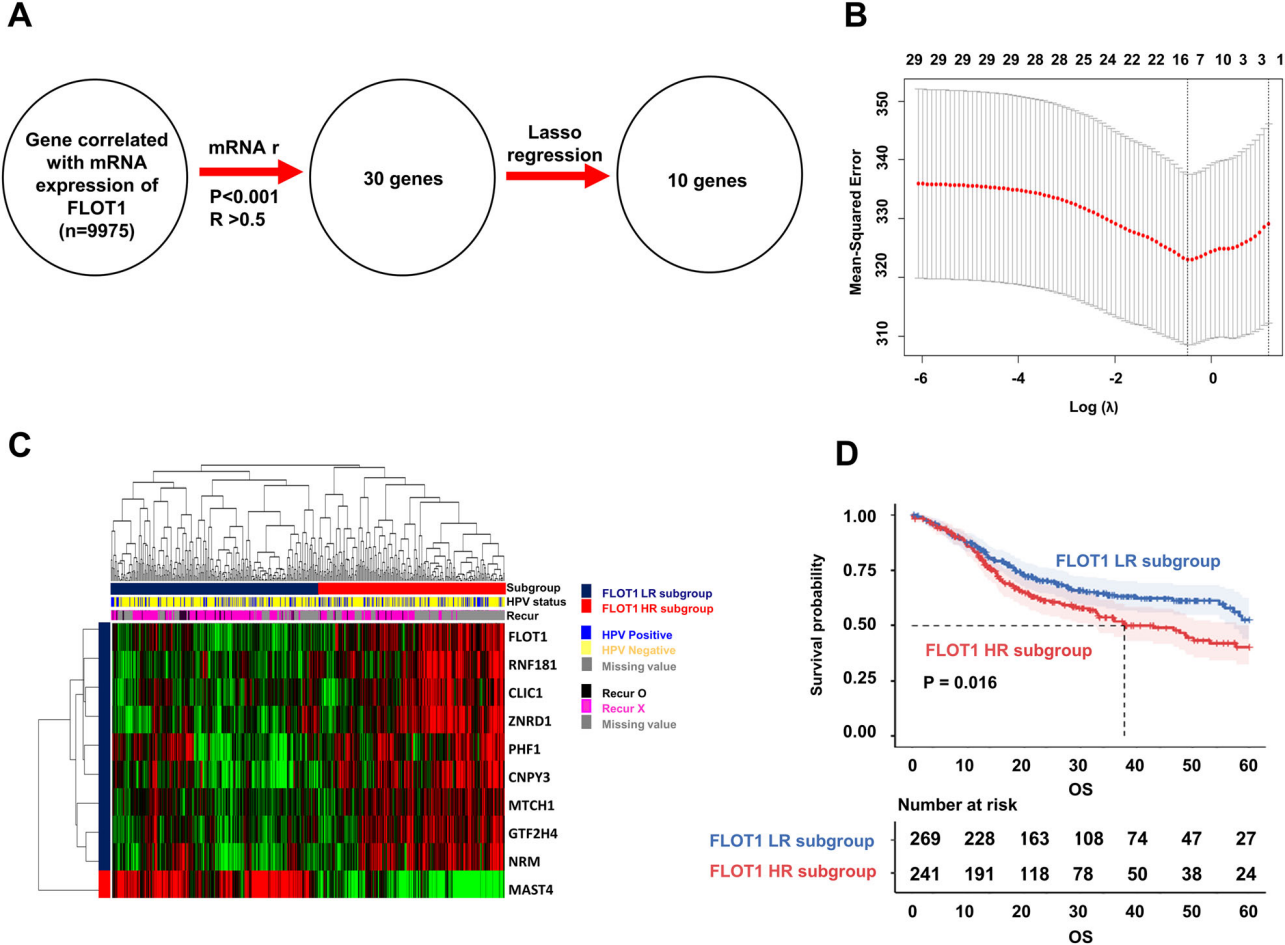
Construction of a FLOT1-related gene signature in head and neck cancer. **A** Overview of the study design for identifying the FLOT1-related gene signature. **B** LASSO coefficient profiles of 10 prognostic genes and cross-validation for tuning parameter (lambda) screening in the LASSO regression model. **C** Unsupervised clustering of the FLOT1-related gene signature in HNSCC. The expression heatmap of FLOT1-related genes shows a clear separation between the FLOT1 HR and FLOT1 LR subgroups. The green and red colors indicate low and high expression, respectively. HPV status and recurrence status are displayed on the bars. In the HPV panel, the yellow color indicates HPV negative, and the blue color indicates HPV positive. In the recurrence panel, the black color indicates patients with recurrence, and the violet color indicates patients without recurrence. The gray color indicates missing values. **D** The FLOT1-related gene signature was significantly associated with OS in TCGA cohort (*P* < 0.05). The significance was calculated using the log-rank test.

Previous studies have reported FLOT1 as a potential marker for HNSCC progression, enhancing the invasion ability of HNSCC cells, and suggesting its role as a biomarker for metastasis [18]. However, its effect on RT in HNSCC patients remains unclear. Additionally, the expression subtypes of FLOT1 (FLOT1 high and low), as determined by the average score, did not significantly predict the prognosis of HNSCC (43.2% vs. 49.9% at 5 years, *P* = 0.161; Supplementary Fig. 1). To explore the molecular profiles of the FLOT1-related gene signature, we systematically analyzed FLOT1-related genes using Pearson’s correlation analysis. The FLOT1-related gene signature is shown in Fig. 1A. Thirty genes were identified whose mRNA expression correlated with FLOT1 gene expression in TCGA cohort; these genes were filtered based on a correlation coefficient (greater than 0.5, or less than −0.5) and a *p* value (less than 0.001). Subsequently, the LASSO regression algorithm was employed to select 10 FLOT1-related genes, determining the penalty coefficient. The minimum log(lambda) was optimized using 10-fold cross-validation (Fig. 1B). Several key genes (*FLOT1*, *RNF181*, *CLIC1*, *ZNRD1*, *PHF1*, *CNPY3*, *MTCH1*, *GTF2H4*, *NRM*, and *MAST4*) were identified. Patients in TCGA cohort were stratified into FLOT1 HR and FLOT1 LR subgroups using this gene signature, and the expression patterns of the FLOT1-related gene signature are depicted in Fig. 1C. Kaplan–Meier analysis and log-rank tests depicted in indicated a significantly lower 5-year OS in the FLOT1 HR subgroup than in the FLOT1 LR subgroup in TCGA cohort (40.3% vs. 52.5% at 5 years, *P* = 0.016; Fig. 1D).

### Validation of FLOT1-related gene signature in three independent cohorts

**Fig. 2:**
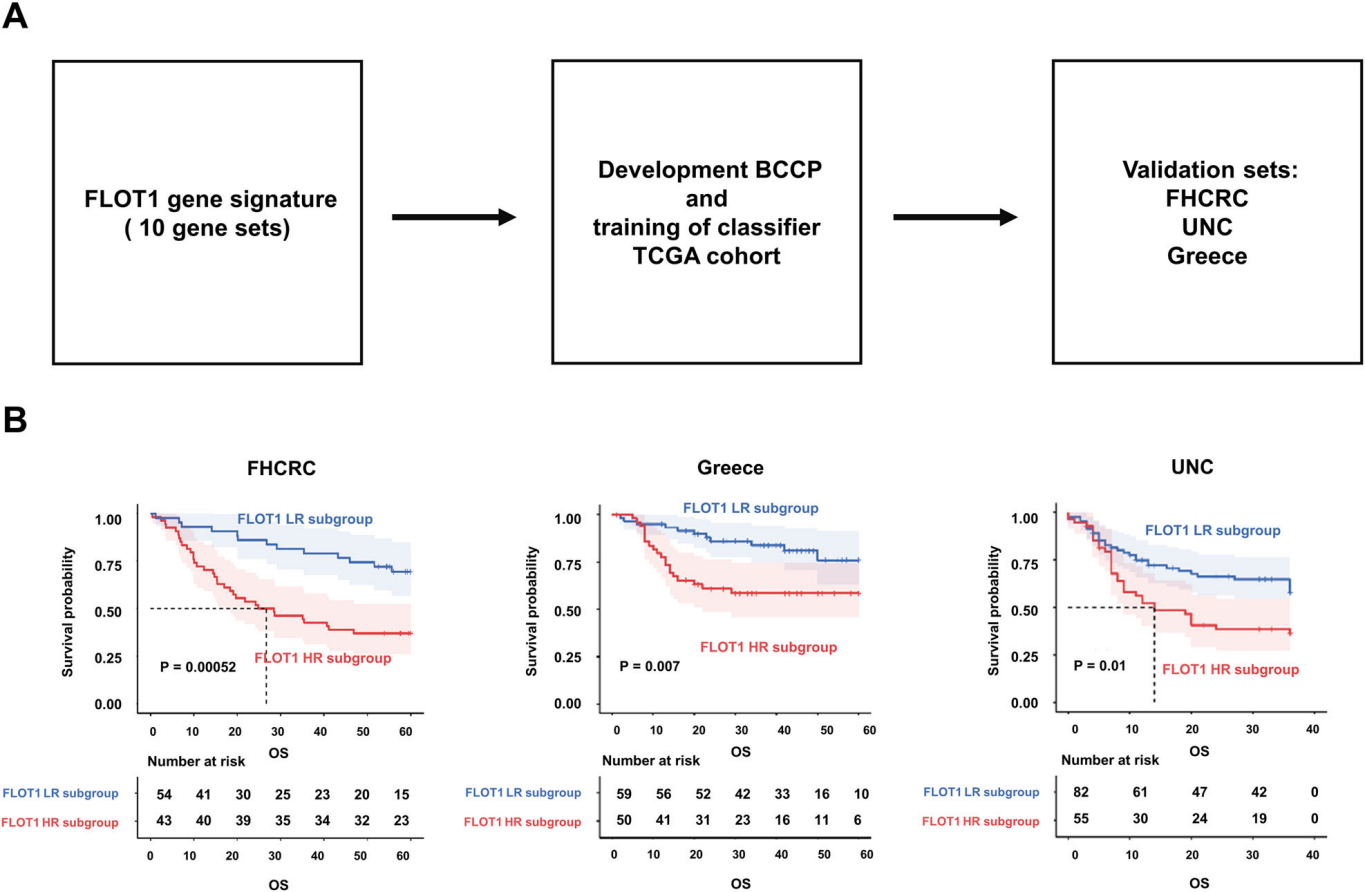
Prognostic performance of the FLOT1-related gene signature in the validation cohorts. **A** A systematic workflow for the validation process in the FHCRC, Greece, and UNC cohorts. **B** The significance of the FLOT1-related gene signature in the validation datasets. The Kaplan–Meier curve shows that the FLOT1 HR subgroup is highly associated with poor prognosis. The significance was calculated using the log-rank test.

We assessed the prognostic significance of FLOT1-related gene signatures in multiple cohorts (Fig. 2A). The FLOT1-related gene signature efficiently classified each of the three independent cohorts into FLOT1 HR and FLOT1 LR subgroups, according to the BCCP classifier, which is consistent with the results obtained for TCGA training dataset. Classification of patients in each test dataset according to the FLOT1-related gene signature showed a poorer prognosis in the FLOT1 HR subgroups than in the FLOT1 LR subgroups. The Kaplan–Meier curves for the FLOT1 HR subgroup patients indicated significantly worse OS durations compared to those in the FLOT1 LR subgroup within the FHCRC cohort (37.0% vs. 69.4% at 5 years, *P* = 0.0042; Fig. 2B). Furthermore, Kaplan–Meier curves revealed markedly poorer RFS rates among FLOT1 HR subgroup patients compared to those in the FLOT1 LR subgroup within the Greek and UNC cohorts (Greece: 58.4% vs. 75.9% at 5 years, *P* = 0.00701; UNC: 36.4% vs. 57.9% at 5 years, *P* = 0.0101).

### FLOT1-related gene signature as a robust independent prognostic factor for HNSCC

**Fig. 3:**
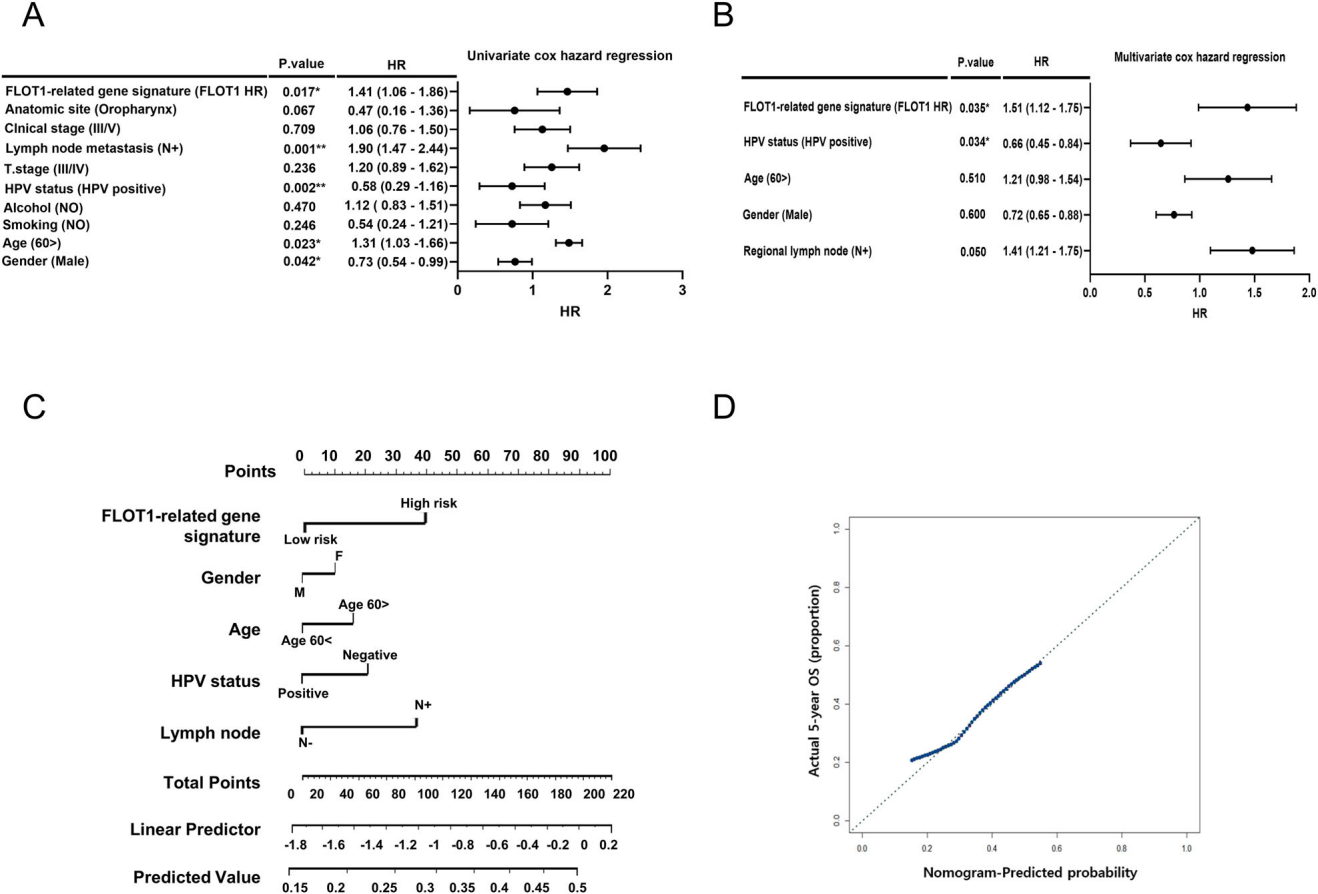
Univariate and multivariate Cox proportional hazard regression analyses in the training set. **A** Forest plot for univariate Cox regression. **B** Forest plot for multivariate Cox regression. **C** A nomogram based on the FLOT1-related gene signature. This nomogram integrates T stage, gender, HPV status, and the FLOT1-related gene signature for OS prediction. **D** ROC curves of OS predictive nomogram in TCGA.

We conducted further analyses to assess whether the prognostic impact of the FLOT1-related gene signature is independent of other clinical variables. Univariate Cox hazard regression revealed that the FLOT1-related gene signature (FLOT1-HR group vs. FLOT1 LR group), sex (male vs. female), HPV status (positive vs. negative), and Clinical T stage (I/II vs. III/IV) were significant prognostic factors for OS in HNSCC (Fig. 3A). Additionally, multivariate Cox proportional hazards regression demonstrated that the FLOT1-related gene signature and HPV status were independent predictors of HNSCC patient survival (Fig. 3B). These findings suggest that the prognostic significance of the FLOT1-related gene signature in HNSCC patients is maintained, even when considering classic clinicopathological prognostic features. Furthermore, we constructed a nomogram model incorporating clinical indices, including the FLOT1-related gene signature, sex, age, HPV status, and regional lymph node metastasis. A higher total score, calculated as the sum of the assigned numbers for each factor in the nomogram, corresponded to lower 5-year OS rates (Fig. 3C). The calibration plot for patient survival prediction indicated good agreement between the nomogram-predicted and actual outcomes (Fig. 3D). “Taken together, these data suggest that our FLOT1-related gene signature may offer valuable prognostic insights for HNSCC patients.”

### Relationship between the FLOT1-related gene signature and advanced tumor stage

We further investigated the correlation between the FLOT1-related gene signature and tumor stage in HNSCC patients. The prognosis of the FLOT1 HR and FLOT1 LR subgroups was evaluated based on tumor stage (tumor stage I-II vs. tumor stage III-IV). Supplementary Fig. 3A illustrates that there were no significant differences in the 5-year OS rates between the FLOT1 HR and FLOT1 LR subgroups in patients with early-stage tumors (36.8% vs. 53.3% at 5 years, *p* = 0.735). However, the FLOT1 HR subgroup exhibited significantly lower 5-year OS rates than the FLOT1 LR subgroup among patients with advanced tumor stages (38.6% vs. 51.6% at 5 years, *P* = 0.00944; Supplementary Fig. 3B).

### The association between FLOT1-related gene signature and response to radiation

**Fig. 4:**
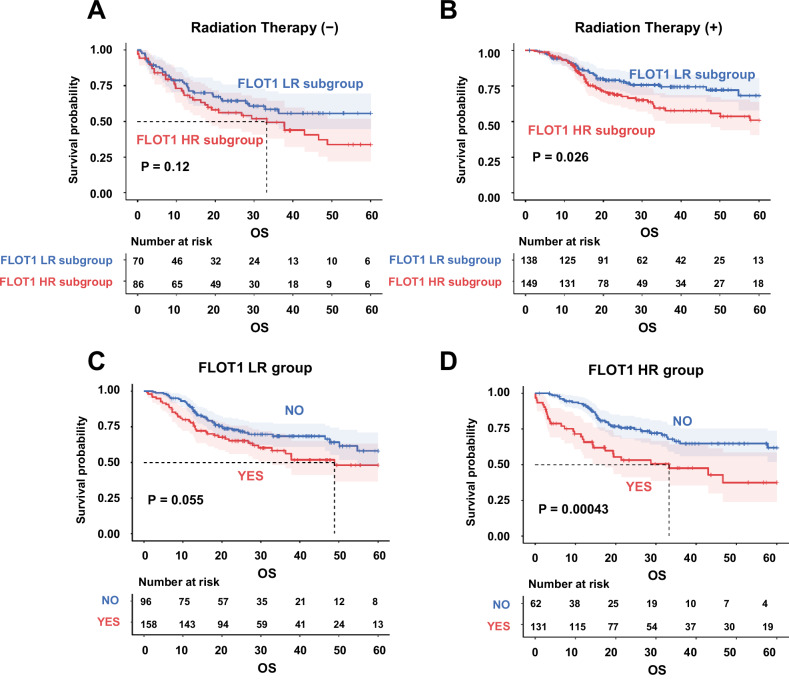
Validation of FLOT1-related gene signature between patients with RT and patients without RT. **A**, **B** The FLOT1-related gene signature significantly predicts the prognosis of RT in HNSCC (*P* < 0.05). The significance was calculated using the log-rank test. **C**, **D** Prediction of the response of patients in the two subgroups to RT according to the FLOT1-related gene signature (*P* < 0.05). Patients in the FLOT1-HR subgroup benefited significantly from RT. The significance was calculated using the log-rank test.

To investigate the association between FLOT1 and the response to RT in HNSCC, we analyzed data from 384 patients obtained from TCGA database. Patients were initially divided into two groups: those who received RT (*n* = 245) and those who did not (*n* = 139). Among the 245 patients undergoing RT, radiation regimen data were available for only 163 patients. Among these, 46 patients received adjuvant treatment, 46 received primary treatment, 2 received neoadjuvant treatment, and 3 received palliative treatment. Data for 66 patients were recorded as not available. For the 163 patients with a recorded radiation dosage, the average dosage was ~5609.65, with a standard deviation of ±1158.23 [19]. For further analysis, the cohort was stratified based on RT status, distinguishing between patients who underwent RT and those who did not. Subsequently, patients were further divided into two groups according to FLOT1-related gene signature subgroups. There were no significant differences in 5-year OS rates between patients who received RT and those who did not in the FLOT1 LR subgroup (40.3% vs. 50.5% at 5 years, *P* = 0.12; Fig. 4A). However, as illustrated in Fig. 4B, the FLOT1 HR subgroup had a significantly worse OS than the FLOT1 LR subgroup among patients who received RT in TCGA cohort (52.7% vs. 65.1% at 5 years, *P* = 0.0261). We then performed an interaction test for OS, which showed a significant correlation between the FLOT1-related gene signature and RT. There were no significant differences in the 5-year OS rates between patients who received RT and those who did not in the FLOT1 LR subgroup (47.6% vs. 55.5% at 5 years, *P* = 0.055; Fig. 4C). However, the prognosis of patients who received RT was better than that of those who did not in the FLOT1 HR subgroup (40.6% vs. 72.7% at 5 years, *P* = 0.000433; Fig. 4D). These results revealed a significant correlation between the FLOT1-related gene expression signature and RT, indicating that patients in the FLOT1 LR subgroup benefited significantly from RT. We then validated the FLOT1-related gene signature in patients, distinguishing between those who received only RT and those who received chemoradiotherapy. The FLOT1-related gene signature significantly predicted the response to both RT and chemoradiotherapy in HNSCC. As shown in Supplementary Fig. 3A, B the FLOT1-HR subgroup was associated with poorer OS and RFS compared to the FLOT1-LR subgroup in HNSCC patients receiving chemoradiotherapy (OS: 43.8% vs. 84.6% at 5 years, *P* = 0.0016; RFS: 28.6% vs. 79.4% at 5 years, *P* = 0.0054, respectively) Additionally, as depicted in Fig. 3C, D the FLOT1-HR subgroup showed worse OS and RFS outcomes in patients receiving only RT compared to the FLOT1-LR subgroup. (OS: 46.0% vs. 75.2% at 5 years, *P* = 0.00025; RFS: 43.0% vs. 79.4% at 5 years, respectively) Thus, FLOT1 may be relevant for the prognosis of HNSCC patients receiving RT. Therefore, we established FLOT1-specific knockdown cells for in vitro analysis.

### The relationship between the FLOT1-related gene signature and HPV status in HNSCC

HPV status is significantly associated with prognosis in patients with HNSCC [20]. Therefore, we analyzed the prognostic outcomes of the FLOT1-LR and FLOT1-HR subgroups in HPV-positive and HPV-negative HNSCC patients from the TCGA database. Kaplan-Meier survival curves revealed significant differences in OS between the FLOT1-LR and FLOT1-HR subgroups in HPV-positive HNSCC patients, with the FLOT1-LR subgroup demonstrating a more favorable prognosis compared to the FLOT1-HR subgroup (66.6% vs. 0.00% at 5 years, *p* = 0.00078, Supplementary Fig. 4A). Furthermore, the FLOT1-related gene signature was predictive of prognosis in HPV-negative HNSCC patients. As shown in Supplementary Fig. 4B, the FLOT1-HR subgroup exhibited significantly lower 5-year OS rates compared to the FLOT1-LR subgroup (36.1% vs. 51.1% at 5 years, *p* = 0.034). These findings suggest that the FLOT1-related gene signature is a prognostic predictor for survival in HNSCC, regardless of HPV status. Next, we investigated the role of the FLOT1-related gene signature in predicting the prognosis of HNSCC patients treated with RT, stratified by HPV status. Next, we investigated the role of the FLOT1-related gene signature in predicting the prognosis of HNSCC patients treated with RT, stratified by HPV status. The OS of patients with RT in the HPV-positive group was shorter in the FLOT1-HR subgroup compared to the FLOT1-LR subgroup. (0.00% vs. 100% at 5 years, *p* = 0.025, Supplementary Fig. 4C). However, there was no significant difference in the OS of HPV-positive patients who did not receive RT between the FLOT1-HR and FLOT1-LR subgroups. (0.00% vs. 44.4% at 5 years, *p* = 0.18, Supplementary Fig. 4D). Additionally, we aimed to determine whether the FLOT1-related gene signature could predict outcomes for HPV-negative patients undergoing RT. In HPV-negative patients receiving RT, the FLOT1-related gene signature significantly predicted OS, with the FLOT1-HR subgroup showing poorer prognosis compared to the FLOT1-LR subgroup. (35.7% vs. 57.5% at 5 years, *p* = 0.011, Supplementary Fig. 4E). In contrast, there was no difference in OS between the FLOT1-HR and FLOT1-LR subgroups for HPV-negative patients who did not receive RT. (41.5% vs. 57.7% at 5 years, *p* = 0.14, Supplementary Fig. 4F). Taken together, the FLOT1-related gene signature is significantly useful for diagnosing HNSCC patients undergoing RT, regardless of HPV status.

### Inhibiting FLOT1 expression enhances HNSCC radiosensitivity via PTEN/IGF1R axis regulation

**Fig. 5:**
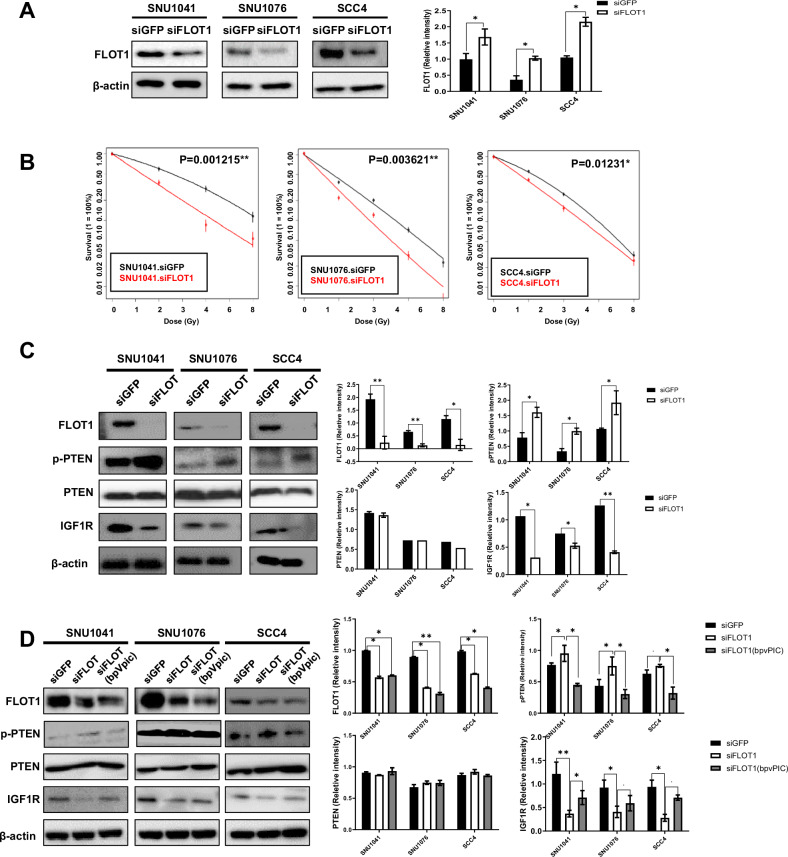
FLOT1 silencing-induced radiosensitivity through the pPTEN/IGF1R axis. **A** Cells were transfected with siGFP (100 µM) as a negative control and siFLOT1 (100 µM). Protein samples were isolated after sorting, and the levels of the indicated proteins were analyzed using western blotting. β-actin was used as a loading control. **B** Clonogenic assays of siGFP and siFLOT1 in SNU1041, SNU1076, and SCC4 cells were performed as described in the Materials and Methods section. Cells were treated with 2, 4, and 8 Gy irradiation. Data points represent the mean surviving fractions (SF) ± the standard deviation (SD) from three independent experiments (*n* = 3; *P* < 0.01*, *P* < 0.01**, and *P* < 0.001*** Two-way ANOVA). Plating efficiency was higher than 12%. **C** FLOT1 regulates radiosensitivity by promoting pPTEN activity and inhibiting IGF1R expression. Cells were transfected with siGFP (100 µM) as a negative control and siFLOT1 (100 µM). Protein samples were isolated after sorting, and the levels of the indicated proteins were analyzed using western blotting. β-actin was used as a loading control. The results in the graphs represent three independent experiments performed in triplicate. P < 0.05, **P < 0.01, and ***P < 0.001, using a two-tailed Student’s *t*-test. **D** Co-suppression of FLOT1 and pPTEN leads to recovery of IGF1R expression. Cells were transfected with siGFP (100 µM) as a negative control and siFLOT1 (100 µM). Twenty-four hours after inhibition of FLOT1 expression, the cells were treated with the PTEN activation inhibitor bpvPIC (5 nM/mL for three HNSCC cell lines), and FLOT1, pPTEN, and IGF1R expression were measured 24 h later. The transfection efficiency was tested after 48 h using western blotting. β-actin was used as a loading control. Results in the graphs represent three independent experiments performed in triplicate. *P* < 0.05, **P < 0.01, and ***P < 0.001, using a two-tailed Student’s *t*-test.

To investigate the role of FLOT1 in radiosensitivity, we applied the FLOT1-related gene signature to the Cancer Cell Line Encyclopedia dataset and stratified cell lines using the BCCP algorithm. In addition, FLOT1 expression was evaluated in multiple HNSCC cell lines. SNU1041, SNU1076, and SCC4 cell lines were classified into the FLOT1 HR subgroup (Supplementary Fig. 5A). Furthermore, these cell lines exhibited markedly higher FLOT1 expression than other cell lines (Supplementary Fig. 5B). Next, we established FLOT1 knockdown cell lines by siRNA transfection. The siFLOT1-transfected cells displayed a significant reduction in the FLOT1 protein level compared to siGFP-transfected SNU1041, SNU1076, and SCC4 cells (Fig. 5A). We investigated whether inhibiting FLOT1 expression affects the radiosensitivity of HNSCC cells using CFA. CFA was performed on the three HNSCC cell lines at irradiation doses of 2, 4, and 8 Gy. The FLOT1-knockdown cells became substantially more sensitive to radiation, with fewer colonies being formed than siGFP cells (Fig. 5B). These data suggest that inhibiting FLOT1 expression promotes radiosensitivity in HNSCC cells. Given that the function of FLOT1 depends on its downstream targets, we investigated the downstream mechanisms associated with FLOT1-related radioresistance. FLOT1 is expressed in lipid rafts, and its ability to compartmentalize different signaling pathways is a key feature of lipid rafts [21, 22]. Moreover, FLOT1 in lipid rafts appears to significantly regulate the initiation of Phosphatase and Tensin Homolog (PTEN) or Insulin-like Growth Factor 1 receptor (IGF1R) signaling pathways by promoting the accumulation of PIP3 in the plasma membrane [23, 24]. Importantly, the activation of PTEN or expression of IGF1R has been reported to play a crucial role in radioresistance [25, 26]. We aimed to explore the radioresistance mechanism underlying the regulation of IGF1R expression by FLOT1 in HNSCC. Therefore, we postulated that FLOT1 contributes to IGF1R expression through phospho-PTEN (pPTEN) suppression. Silencing FLOT1 suppressed IGF1R expression and activated PTEN expression (Fig. 5C). These results indicated that FLOT1 is a major regulator of PTEN and IGF1R in HNSCC cells. Next, we explored the PTEN/IGF1R axis-related radiation regulation. First, we knocked down FLOT1 expression in HNSCC cells using siRNA. Following the depletion of FLOT1 in HNSCC cells, treatment with bpvPIC, a suppressor of PTEN activation, was administered. The concentration of bpvPIC (5 nM/mL) was determined based on their effect on cell viability (Supplementary Fig. 6). Co-inhibition of the expression of the target genes resulted in FLOT1 and PTEN downregulation, while notably restoring IGF1R expression (Fig. 5D). These results provided evidence that FLOT1 mediates the function of pPTEN/IGF1R in the regulation of radioresistance in HNSCC.

### Inhibiting FLOT1 Expression Induces HNSCC Cell Death under Radiation

**Fig. 6:**
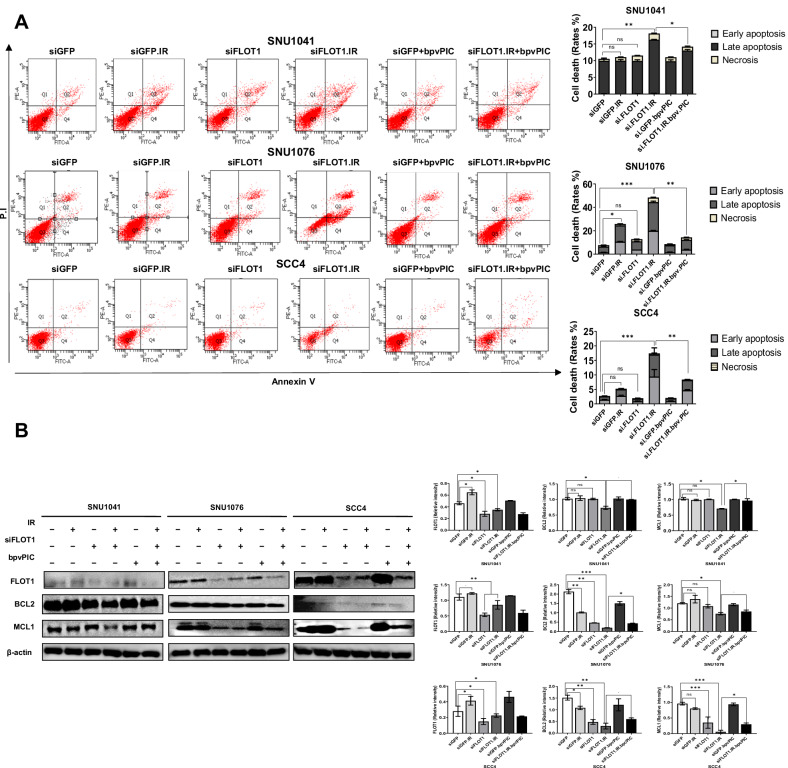
Inhibition of FLOT1 expression triggers the cell death under radiation in HNSCC cells. **A** Knockdown of FLOT1 expression enhanced the apoptosis of SNU1041, SNU1076, and SCC4 cells. Cells were transfected with siGFP (100 µM) as a negative control and siFLOT1(100 µM), and 24 h after inhibition of FLOT1 expression, cells were treated with the PTEN activation inhibitor bpvPIC (5 nM/mL for three cell lines). Twenty-four hours after inhibition of FLOT1 expression, cells were irradiated at a dose 4 Gy of IR. Annexin V staining was performed to measure cell death rates at least three independent experiments. (*P* < 0.05, **P < 0.01, and ***P < 0.001, by two-tailed Student’s *t*-test). **B** Knockdown of FLOT1 under RT exposure downregulated the expression of anti-apoptotic proteins BCL2 and MCL1 in SNU1041, SNU1076, and SCC4 cells in vitro. Protein samples were isolated, and the level of proteins was analyzed using western blotting. β-actin was used as loading control. Results in the graphs represent three independent experiments performed in triplicate. Data represent the mean ± SD. *P < 0.05, **P < 0.01, and ***P < 0.001, using a two-tailed Student’s *t*-test.

Compartmentalization of death receptors and downstream signaling in lipid rafts can initiate apoptosis. In this context, the IGF1R has been identified as the most potent activator of cell death through modulation of the BCL2/MCL1 pathway [21, 22]. To investigate whether FLOT1 regulates radiation-induced apoptosis and apoptosis-related proteins, the expression levels of cell death and anti-apoptotic proteins BCL2 and MCL1 were evaluated. Following the combination of FLOT1 depletion and IR, cell death (%) and MCL1 and BCL2 expression levels were measured. After FLOT1 knockdown, there was a significant increase in HNSCC cell death 48 h following exposure to 4 Gy of irradiation, and the expression levels of MCL1 and BCL2 were decreased compared to siGFP-treated cells (*P* < 0.05; Fig. 6A, B). Thus, FLOT1 may play a role in HNSCC cell survival and resistance to irradiation-induced apoptosis. Additionally, the use of bpvPICs at a concentration of 5 nM/ml was able to rescue the cells from death, possibly through its inhibitory effect on p-PTEN (*P* < 0.05; Fig. 6A, B). This was supported by the observed increase in the expression of MCL1 and BCL2, which are known to promote cell survival.

### Confirming the efficacy of FLOT1 as a radiosensitizer in radioresistant cells

**Fig. 7:**
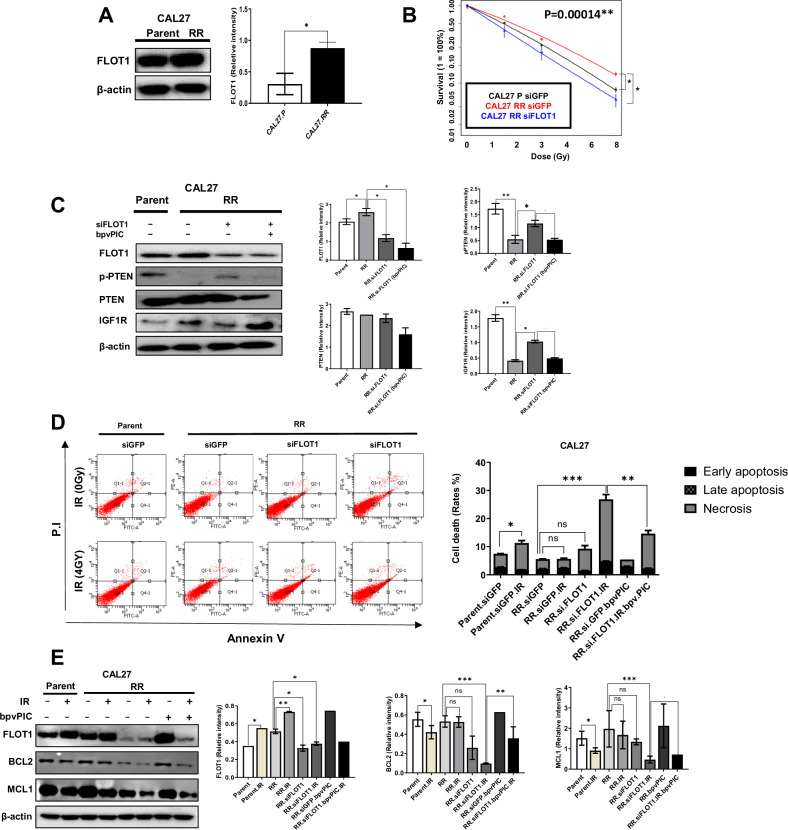
Restoration of radiosensitivity in radioresistant HNSCC cells after inhibiting FLOT1 expression. **A** Western blot analysis was used to determine FLOT1 protein levels in CAL27-P and CAL27-RR cell lines. **B** CAL27-P and CAL27-RR cells transfected with siGFP or siFLOT1 were exposed to radiation doses of 2, 4, and 8 Gy, followed by CFA performed in triplicate. Data points represent the mean surviving fractions (SF) ± standard deviation (SD) from three independent experiments (*n* = 3; *P < 0.05, **P < 0.001, ***P < 0.0001, Two-way ANOVA). Plating efficiency was higher than 12%. **C** CAL27-P and CAL27-RR cells transfected with siGFP or siFLOT1 were evaluated for levels of FLOT1, pPTEN, and IGF1R proteins. In CAL27-RR cells, siFLOT1-transfected cells were also treated with bpvPIC (5 nM/mL). Protein levels were determined using western blotting. **D** Cell apoptosis was assessed using Annexin V-FITC/PI staining followed by flow cytometry analysis. CAL27-P-siGFP, CAL27-RR-siGFP, and CAL27-RR-FLOT1 cells were exposed to 0 or 4 Gy radiation and analyzed using an Annexin V-FITC Apoptosis Detection Kit. All experiments were performed in triplicate. Two-way ANOVA was used to estimate the *P* value (*P* < 0.05). **E** CAL27-P and CAL27-RR cells transfected with siGFP or siFLOT1 were exposed to radiation doses of 0 or 4 Gy, and the levels of FLOT1, MCL1, and BCL2 proteins were analyzed using western blotting. **A**, **C**, **E** β-Actin served as an internal loading control. Graphs represent three independent experiments performed in triplicate. Data represent the mean ± SD. *P < 0.05, **P < 0.01, and ***P < 0.001, by two-tailed Student’s *t*-test.

To further investigate whether inhibiting FLOT1 expression in radioresistant HNSCC cells modulates radiosensitivity, we exposed an HNSCC cell line to repeated 2 Gy doses of RT over 15 weeks, resulting in the formation of a line that was significantly more radioresistant (CAL27-RR) than the parental cell from which it was derived (CAL27-P) [23]. The expression levels of FLOT1 were significantly higher in CAL27-RR cells than in CAL27-P cells (Fig. 7A). Subsequently, CAL27-RR cells were transfected with either siFLOT1 or siGFP, and survival rates were assessed using CFA. The survival rate of CAL27-RR cells was significantly higher than that of CAL27-P cells after IR exposure (Fig. 7B). However, when FLOT1 was depleted using siRNA constructs, a significant increase in the radiosensitivity of CAL27-RR cells to IR was observed, indicating that the combination of FLOT1 depletion and RT reduces radiation resistance. Additionally, we attempted to verify that FLOT1, a key factor that regulates radioresistance, mediates the p-PTEN/IGF1R axis in CAL27-RR cells. Compared to CAL27-P cells, CAL27-RR cells exhibited decreased p-PTEN levels, which increased upon the inhibition of FLOT1 expression (Fig. 7C). In addition, CAL27-RR cells exhibited increased IGF1R expression compared to CAL27-P cells, which subsequently decreased upon the inhibition of FLOT1 expression. Furthermore, we confirmed the p-PTEN and IGF1R levels after the co-inhibition of FLOT1 and PTEN expression in CAL27-RR cells. Co-inhibition of FLOT1 and PTEN expression using siFLOT1 and bpvPIC induced the downregulation of p-PTEN and PTEN expression compared to the inhibition of FLOT1 expression alone in CAL27-RR cells. Conversely, co-inhibition of FLOT1 and PTEN expression led to the upregulation of IGF1R, compared to the inhibition of FLOT1 expression alone. These results indicated that FLOT1 regulates IGF1R expression by modulating PTEN activity in CAL27-RR cells. We then examined the impact of FLOT1 and the PTEN/IGF1R axis in CAL27-RR cells under IR irradiation. As shown in Supplementary Fig. 7, following IR, FLOT1 and p-PTEN expression were increased, while IGF1R expression was decreased in CAL27-P cells. FLOT1 and p-PTEN expression were enhanced, while IGF1R expression was decreased in CAL27-RR cells compared to CAL27-P cells. However, there were no significant differences in the expression of FLOT1, p-PTEN, and IGF1R between CAL27-RR cells and CAL27-RR cells treated with IR. However, when FLOT1 was inhibited. FLOT1 and IGF1R expression were downregulated, whereas p-PTEN expression was increased. These changes became dramatically pronounced under IR irradiation. Furthermore, co-inhibition of FLOT1 and PTEN expression following IR led to downregulation of p-PTEN, while simultaneously causing a significant upregulation of IGF1R expression. These findings suggest that RT may enhance the regulatory effects of FLOT1 and PTEN on the p-PTEN/IGF1R axis. Subsequently, we examined the effects of FLOT1 inhibition combined with RT on the cell death rates in CAL27-RR cells. While the exposure of CAL27-P cells to 4 Gy of IR resulted in increased cell death rates compared to non-irradiated cells, no significant differences were observed in CAL27-RR cells exposed to the same dose (Fig. 7D). Moreover, a single treatment with siFLOT1 in CAL27-RR cells did not result in a significant increase in cell death. However, the combination of FLOT1 depletion and irradiation dramatically increased the death rate of CAL27-RR cells. These effects were attenuated by bpvPICs at a concentration of 5 nM. Consistently, exposure to 4 Gy of IR reduced the levels of the anti-apoptotic molecules MCL1 and BCL2 in CAL27-P cells, compared with those in the non-irradiated cells (Fig. 7E). However, no significant differences were observed between CAL27-RR and CAL27-RR cells treated with 4 Gy of IR. In addition, no significant difference was observed in the expression of MCL1 and BCL2 in CAL27-RR cells after the inhibition of FLOT1 expression. In contrast, the suppression of FLOT1 expression combined with IR dramatically diminished MCL1 and BCL2 expression in CAL27-RR cells. However, the expression of MCL1 and BCL2 increased after treatment with bpvPICs. These results indicated that FLOT1 knockdown markedly restored radiosensitivity in radioresistant cancer cells.

### Inhibiting FLOT1 expression increases radiosensitivity efficacy in xenograft model

**Fig. 8:**
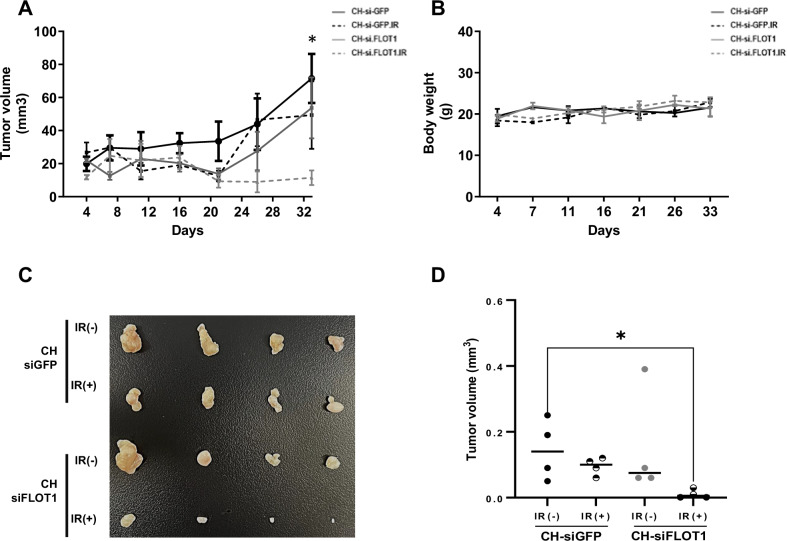
Inhibiting FLOT1 expression enhances radiosensitivity in HNSCC xenograft mouse model under irradiation exposure. **A** CAL27-RR tumor-bearing mice were administered CH loaded with siGFP (CH-siGFP) or siFLOT1 (CH-siFLOT1) with or without RT. Tumor volume was measured twice a week, (**B**) Mouse weights were recorded at identical intervals. **C** Representative photographs of dissected tumors are shown. **D** Tumor weights were measured in the indicated groups (*n* = 8; *P < 0.05, **P < 0.01, and ***P < 0.001; two-way ANOVA).

Based on our in vitro findings, we hypothesized that the combination of RT with inhibition of FLOT1 expression could reverse radioresistance in a mouse model. To investigate this, xenograft experiments were conducted in nude mice by injecting 2 × 10^6^ CAL27-RR cells into their thighs. CAL27-RR cells exhibited radioresistance compared to CAL27-P cells in a mouse model (data not shown). Eight BALB/c nude mice were randomized into four treatment cohorts: CH-siGFP, CH-siGFP with IR, CH-siFLOT1, and CH-siFLOT1 with IR. Three days after tumor implantation, the CH loaded with siRNAs (siGFP and siFLOT1) was administered subcutaneously into the tumor site. Three days after CH loading, 10 Gy of RT was administered to the xenograft tumors in 2 Gy fractions over 5 days. Tumor growth was monitored for up to 33 days post IR to assess the combined effect of FLOT1 depletion and IR on tumor growth in vivo. Consequently, tumor growth in mice treated with both CH siFLOT1 and IR was significantly decreased compared to that in the single treatment groups with CH siGFP, siFLOT1, or siGFP with IR alone from day 21 onwards (Fig. 8A). Conversely, when treated with RT or CH siFLOT1 alone, no significant difference in tumor growth was observed compared with CH siGFP-treated tumors. These results demonstrate that combined treatment with RT and CH siFLOT1 effectively inhibited tumor growth in the mouse model (*P* < 0.05). Furthermore, body weight changes have been reliably used as powerful indicators to study the toxicity of injected materials in mouse models. The body weight of BALB/c mice showed no significant changes throughout the 33-day experiment (Fig. 8B). After 33 days, the mice were euthanized, and the tumor volume and weight were measured. Treatment with CH siGFP and RT or CH siFLOT1 did not significantly alter tumor volume and weight compared with CH siGFP (*P* > 0.05; Fig. 8C, D). However, the combination of CH siFLOT1 and RT significantly decreased the tumor volume and weight compared to CH siGFP (*P* < 0.05). To assess the effect sizes in the in vivo studies and account for small sample bias, we computed Hedges’ g [24]. The results showed that the effect sizes were: CH-siGFP vs CH-siGFP with IR = 1.91, CH-siGFP vs CH-siFLOT1 = 0.17, and CH-siGFP vs CH-siFLOT1 with IR = 2.34. The combination of CH-siGFP and CH-siFLOT1 showed medium effects. In contrast, single treatments of CH-siFLOT1 or CH-siGFP with IR showed small effects compared to CH-siGFP. (Supplementary Fig. 8) These findings suggest that FLOT1 might contribute to resistance against RT and that combined treatment with RT and siFLOT1 overcomes radioresistance in an in vivo model.

## Discussion

Radio-resistance remains one of the key challenges in the treatment of HNSCC, significantly impacting therapeutic outcomes. Enhancing the radiosensitivity of HNSCC cells holds great potential for improving patient survival rates. In response to this challenge, recent studies have increasingly focused on developing predictive tools, identifying biomarkers, and discovering gene signatures to overcome radio-resistance in HNSCC [25–28]. In our study, we emphasized the significance of FLOT1 in predicting prognosis and radioresistance in HNSCC. By identifying FLOT1-related genes, we developed an optimized gene signature through Lasso regression analysis. This approach is unique in that it pinpoints a refined set of genes specifically associated with FLOT1, offering a targeted strategy to address radioresistance in HNSCC. Then, two distinct subgroups were identified based on the FLOT1-related gene signature: the FLOT1 HR subgroup and the FLOT1 LR subgroup. Findings from four independent cohorts of HNSCC patients underscore the clinical relevance of FLOT1, with HR patients exhibiting poorer survival outcomes than their LR counterparts. Our analysis revealed a strong association between the FLOT1-related gene signature and response to RT, further highlighting FLOT1’s role in radioresistance mechanisms in HNSCC cells.

Inhibition of FLOT1 led to a decrease in radioresistance. Mechanistically, we found that inhibiting FLOT1 expression upregulated pPTEN expression, which subsequently decreased IGF1R expression in HNSCC. Moreover, our study demonstrated that FLOT1 knockdown enhanced radiosensitivity by promoting apoptosis, further highlighting the critical role of FLOT1 in regulating cell survival following radiation exposure. In a xenograft model, the combination of CH siFLOT1 with IR resulted in a significant reduction in tumor growth compared to either treatment alone, reinforcing the efficacy of targeting FLOT1 to enhance the therapeutic response to RT. This study presents a significant advancement over previous research by exploring the clinical significance and molecular mechanisms of FLOT1 in the context of radioresistance.

The flotillin protein family, also known as reggies, encompasses two homologous isoforms, FLOT1 and FLOT2, which reside in lipid rafts [29, 30]. Flotillin proteins form heterooligomeric complexes that facilitate various cellular processes, such as cell adhesion, actin cytoskeleton reorganization, endocytosis, and signal transduction [31–35]. Their signaling transduction is initiated by growth factors and membrane-resident receptor kinases [36]. Studies have implicated flotillins in tumorigenesis, with FLOT1 notably involved in crucial pathways like TGF-β signaling, impacting EMT [8]. FLOT1 is upregulated in various malignancies, including endometrial, cervical, renal, breast, lung, nasopharyngeal, and esophageal cancers, where it functions as an oncogene [15, 17, 35, 37–39]. In gastric cancer, FLOT1 is involved in tumor progression and metastasis by regulating EMT signaling. This progression and metastasis have been reported to be associated with the stabilization of Snail and the activation of the BCAR1/ERK signaling pathway [17, 40, 41]. Furthermore, silencing FLOT1 inhibits proliferation and tumorigenicity in breast cancer cells by upregulating FOXO3a [14]. Moreover, FLOT1 mediates the levels of heterotypic signaling molecules, including TNF-α, EGF, HGF, and IGF1, across various cancers, such as breast, prostate, and gastric cancer, and oral squamous cell carcinoma [9, 15, 17, 35, 42]. Upregulation of FLOT1 has been associated with poor prognosis in these cancers, indicating its involvement in proliferation and metastasis [12, 18]. In HNSCC, as observed in other malignancies, the expression of FLOT1 is clinically associated with poor prognosis. Moreover, FLOT1 has been shown to promote cancer cell migration and invasion in HNSCC cells [43, 44]. Although previous studies have explored the role of FLOT1 in tumor progression and invasion, its potential involvement in mediating radiation resistance remains unexplored. Furthermore, in HNSCC, the mechanism of FLOT1 remains unclear, and its role in malignancy-associated processes has not yet been fully elucidated. In this study, we investigated the potential of FLOT1 as a prognostic biomarker for HNSCC after RT and explored the mechanisms associated with radioresistance. Given the predominant localization of FLOT1 in lipid rafts and its regulation of downstream signaling, relying solely on FLOT1 as a single biomarker may not be sufficient to accurately predict the prognosis and response to RT in HNSCC. Thus, we systematically analyzed genomic data from HNSCC patients and found that gene sets associated with FLOT1 accurately predicted prognosis. To validate the potential role of FLOT1 in HNSCC radioresistance, we conducted in vitro and in vivo experiments. Additionally, we examined the expression patterns of downstream genes targeted by FLOT1 in HNSCC cells to assess their activation status during signal transduction. Our study focused on the activation of PTEN, a tumor suppressor regulated by lipid raft activation and implicated in cellular processes such as growth, survival, and metabolism [45–47]. PTEN is synthesized in the nucleus and is closely associated with the cell membrane. It has been reported that PTEN is phosphorylated by kinase enzymes, which then plays a key role in the inhibition of the PI3K-AKT signaling pathway. The activation of PTEN primarily involves the dephosphorylation of PIP3 (Phosphatidylinositol-3,4,5-trisphosphate) to PIP2 (Phosphatidylinositol-4,5-bisphosphate) at the cell membrane, thereby inhibiting the PI3K/AKT signaling and cancer-promoting pathways [48, 49]. This process is known to suppress cell growth, survival, and migration [50]. Therefore, loss-of-function mutations in PTEN have been associated with radioresistance in colorectal cancer, poor prognosis, and resistance to chemotherapy in prostate cancer [51–53]. Additionally, we considered the major radioresistance mechanism of the IGF1R, which is dependent on lipid raft activation [54]. IGF1R, a major inhibitor of apoptosis in cancer cells, contributes to metastatic tumor growth and radioresistance [55]. Research indicates that FLOT1 palmitoylation in the endoplasmic reticulum (ER) is necessary for IGF1R transport, which impacts prostate cancer cell proliferation [9, 30, 56]. The association between FLOT1 and the IGF1R triggers anti-apoptotic signaling pathways, suggesting their role in activating signaling pathways in human cancers [57, 58]. Furthermore, previous studies have suggested that FLOT1 localized in lipid rafts initiates the PTEN or IGF1R signaling pathways by enhancing the accumulation of PIP3 in the plasma membrane [45, 47, 59]. Therefore, we hypothesized that FLOT1 regulates radioresistance by downregulating PTEN and upregulating IGF1R. As expected, inhibiting FLOT1 expression downregulated IGF1R and upregulated p-PTEN. Next, we attempted to elucidate the mechanisms underlying the relationship between p-PTEN and IGF1R; suppressing PTEN activation was found to recover IGF1R expression. These findings demonstrate that inhibiting FLOT1 expression induces radiation sensitivity in HNSCC cells by targeting p-PTEN expression and subsequently downregulating IGF1R expression. In addition, because the PTEN/IGF1R axis has been implicated as a major modulator of the development of radioresistance and apoptosis [52, 60], we propose that inhibiting FLOT1 expression may be an effective strategy to overcome radioresistance in HNSCC by enhancing apoptosis. Indeed, the combination of RT and inhibiting FLOT1 expression dramatically increased apoptosis and decreased the expression of anti-apoptotic molecules (BCL2 and MCL1). We found that FLOT1 is associated with radioresistance and poor prognosis in HNSCC patients and that FLOT1 expression influences radioresistance by regulating the pPTEN/IGF1R axis. Thus, FLOT1 can be used as a predictive biomarker, enabling the selection of patients who will benefit from the combination of RT and inhibiting FLOT1 expression.

In conclusion, we identified and validated a novel FLOT1 related signature that facilitates the identification of HNSCC patients with poor prognosis and radioresistance. Furthermore, our results provide a strong rationale for inhibiting FLOT1 expression as a promising strategy, particularly for radiation-based cancer therapy.

### Limitations of the study

Despite the significant radiosensitizing effect of FLOT1 observed in this study, further investigations are required to facilitate its clinical translation for HNSCC treatment. There is a need to comprehensively evaluate the radiosensitizing roles of PTEN and IGF1R in HNSCC, as their precise contributions remain to be fully elucidated. Furthermore, the regulatory interplay between PTEN, IGF1R, and cell death mechanisms must be clearly delineated to establish a mechanistic framework that underpins their role in tumor response to RT.

Another limitation of this study is the relatively small sample size in the validation cohorts, which may impact the validity and statistical power of our findings. Furthermore, although in vivo mouse models demonstrated a synergistic effect in the FLOT1 inhibition and RT combination group, the limited number of mice may reduce the statistical robustness of the results. Future studies with larger sample sizes are needed to validate these observations and enhance the robustness of our conclusions.

In addition, differences in RT dose and regimen among the independent cohorts, where the doses are not clearly defined, may affect the results. While most RT regimens and doses generally adhere to established international guidelines, the lack of clear information on the regimen and dose in some cohorts may slightly reduce the accuracy of our validation. We acknowledge that the impact of these differences was not fully evaluated and remains a limitation of our study.

## Material and methods

### Gene expression and clinical database

Gene expression and clinical data from TCGA (*n* = 566) were downloaded from the UCSC Cancer Genomics Browser (https://xena.ucsc.edu/public). Data from the Fred Hutchinson Cancer Research Center (FHCRC cohort, GSE41613, *n* = 97), AHEPA Hospital in Thessaloniki (Greece cohort, GSE27020, *n* = 109), and UNC CHAPEL HILL (UNC cohort, GSE39368, *n* = 138) were downloaded from the National Center for Biotechnology Information Gene Expression Omnibus database (http://www.ncbi.nlm.nih.gov/geo). Patient characteristics are described in Table 1.

**Table 1:** Clinical and pathological features of HNSCC in 4 cohorts.

	TCGA cohort	Greece	UNC	FHCRC cohort
Number of patients	520	109	138	97
Gender				
Male	384 (73.8%)	104	95 (68.8%)	66 (68.0%)
Female	136 (26.2%)	5	43 (31.2%)	31 (32.0%)
Age (mean ± SD)	62.2 ± 15.3	64.0 ± 9.81	57.0 ± 12.2	NA
Anatomic site				
Oral cavity	315 (60.5%)	NA	55 (39.9%)	86 (88.7%)
Oropharynx	79 (15.1%)	NA	34 (24.6%)	11 (11.3%)
Larynx	116 (22.3%)	NA	30 (21.8)	0
Hypopharynx	10 (1.9%)	NA	13 (9.4%)	0
Others			6 (4.3%)	0
Primary tumor				
T1	35 (6.8%)	NA	13 (9.4%)	NA
T2	151 (29.4%)	NA	27 (19.6%)	NA
T3	132 (25.8%)	NA	25 (18.1%)	NA
T4	195 (38.0%)	NA	52 (37.7%)	NA
Regional lymph node				
N0	244 (48.1%)	NA	51 (37.0%)	NA
N1	83 (16.4%)	NA	15 (10.9%)	NA
N2	162 (32.0%)	NA	46 (33.3%)	NA
N3	18 (3.5%)	NA	5 (3.6%)	NA
Stage				
I	20 (4.0%)	12	10 (7.2%)	30 (30.9%)
II	98 (19.3%)	18	14 (10.1%)	22 (11.3%)
III	105 (20.7%)	36	28 (20.3%)	26 (15.5%)
IV	283 (56.0%)	43	84 (6.9%)	52 (42.3%)
HPV status				
Positive	68 (19.9%)	NA	14 (10.1%)	0
Negative	274 (80.1%)	NA	82 (59.4%)	97 (100%)
Tobacco use				
Never	114 (23.3%)	51	27 (19.6%)	NA
Yes	376 (76.7%)	58	109 (79.0%)	NA
Alcohol use				
Never	162 (31.8%)	1	86 (62.3%)	NA
Yes	347 (68.2%)	108	50 (36.2%)	NA
FLOT1 related gene signature				
Cluster 1	270 (51.9%)	59	83 (60.1%)	54 (55.6%)
Cluster 2	243 (46.7%)	50	55 (39.9%)	43 (44.3%)

### Identification and validation of FLOT1-related gene signature

To develop FLOT1‐related gene signature in HNSCC, gene expression was analyzed from TCGA cohort. We specifically identified genes whose mRNA expression levels were either positively or negatively correlated with FLOT1 expression using a Pearson correlation analysis (|r| > 0.5, *P* < 0.001). Next, FLOT1-related genes were screened using LASSO regression (glmnet R package). Consequently, ten FLOT1-related genes were identified. We performed unsupervised hierarchical clustering analysis with the uncentered correlation coefficient as a measure of similarity and a complete linkage clustering method using the Cluster 3.0 program (Stanford University, Stanford, CA, USA; downloaded at https://cluster2.software.informer.com). HNSCC patients were divided into two subgroups: FLOT1 High-Risk (HR) subgroup and FLOT1 Low-Risk (LR). To further validate the FLOT1-related gene signature in other independent cohorts, all gene expression data for each cohort were standardized by transformation to a median of 0 and a standard deviation of 1. The Bayesian compound covariate predictor (BCCP) class prediction engine was used to test the ability of the FLOT1-related gene signature to predict the class of HNSCC patients in three independent cohorts. Gene expression data from TCGA cohort were combined to form a series of classifiers according to the BCCP algorithm, following which the robustness of the classifier was estimated according to the misclassification rate determined during leave-one-out cross-validation of the training set using BRB-Array Tools [19]. The validation was conducted in three independent cohorts (FHCRC, Greece, and UNC).

### Construction of the nomogram

Univariate and multivariate Cox regression analyses were performed to evaluate whether the risk score could serve as an independent prognostic indicator in patients, univariate and multivariate Cox regression analyses were performed. We utilized data from 520 patients with clinicopathological parameters from TCGA database. Parameters such as FLOT1-related gene signature, sex, age, HPV status, and regional lymph node involvement were included in the analysis. A nomogram was constructed to visualize multivariate Cox regression. Variables with a *P* value < 0.05 in the multivariate Cox regression were incorporated into the nomogram using the R package “rms” to predict 5-year Overall Survival (OS) rates.

### Cell culture and transfection

The SNU1041 cell line was provided by Prof. Chul Ho Kim (Aju University). The SNU1076 cell line was purchased from the Korean Cell Line Bank (KCLB), while SCC4 and CAL27 cell lines were purchased from the American Type Culture Collection (ATCC). SNU1041 and SNU1076 cells were cultured in the RPMI medium. (Corning, Manassas, VA, USA) SCC4 cells were cultured in DMEM/F12 medium (Corning). CAL27 cells were cultured in DMEM/HIGH GLUCOSE medium (Corning). All cells were supplemented with 10% Fetal Bovine Serum (FBS, Corning) and 1% penicillin-streptomycin (PS, Corning), and maintained in media specific to each cell line at 37 °C in a humidified atmosphere containing 5% CO_2_. All cell lines were purchased within the last 5 years, and their identities were confirmed by short tandem repeat (STR) profiling using KCLB. The siRNAs specific for FLOT1 (5′-rCrCrC rUrCrA rArUrG rUrCrA rArGrA rGrUrG rArArA rArGG T-3′) were designed and synthesized by IDT (Cambridge, MA, USA). siRNAs specific to GFP (5′-GCAUCAAGGUGAACUUCAA-3′) were obtained from Bioneer (Daejeon, Korea). Transfection of control-siGFP and FLOT1-siRNA was performed using Lipofectamine RNAiMax (Invitrogen), following the manufacturer’s instructions. Knockdown of FLOT1 was further confirmed by western blotting.

### MTT Assay

To evaluate the effect of bpvPICs on cell viability, MTT assays were conducted using an EZ-Cytox Cell Viability Assay Kit (Daeli Lab Service, Seoul, Republic of Korea). HNSCC cells were seeded at a density of 5000 cells/well in 96-well plates and treated with bpvPICs (1.25, 2.5, 5, 10, 20, and 40 nM/ml). Following a 24-h incubation period in a humidified atmosphere containing 5% CO_2_ at 37 °C, cell viability was assessed using the MTT assay, with absorbance measured at 570 nm. bpvPIC was purchased from Merck (Darmstadt, Germany).

### Colony formation assay

A colony forming assay (CFA) was performed to assess the ability of cells to form colonies. GFP or FLOT1-specific siRNA-treated cells were plated in 6-well plates at a density of 300 cells per well in growth medium supplemented with 10% FBS and 1% PS, then incubated at 37 °C. After 24 h, the cells were irradiated with 0, 2, 4, or 8 Gy radiation. Following irradiation, all cells were maintained at 37 °C for 10–14 d. The cells were then stained with crystal violet for 10 min at room temperature. All experiments were conducted in triplicate. The significance of the differences between dose responses was determined using two-way ANOVA.

### Western blotting

HNSCC cells were lysed in RIPA buffer (50 mM Tris/HCl, 150 mM NaCl, 2 mM EDTA, and 1% Triton™ X-100) supplemented with protease inhibitors (Roche, Mannheim, Germany) and phosphatase inhibitors (Sigma-Aldrich, Burlington, MA, USA). Protein concentrations were determined using the BCA protein assay kit (Thermo Fisher Scientific, Waltham, MA, USA). For western blotting, 15 μg of protein was mixed with SDS sample buffer (Invitrogen), boiled for 10 min at 100 °C, and electrophoresed on an 8–15% gradient bis-Tris gel. Subsequently, the proteins were transferred onto polyvinylidene fluoride membranes (Millipore, Billerica, MA, USA). After blocking the membrane with Tris-buffered saline (TBS) containing 5% nonfat dry milk, it was incubated overnight at 4 °C with the following primary antibodies: anti-FLOT1 (1:1000; Cell Signaling), anti-PTEN (1:1000; Santa Cruz Biotechnology, Santa Cruz, CA, USA), anti-IGF1R (1:1000; Santa Cruz Biotechnology), anti-pPTEN (1:1000; Cell Signaling Technology, Danvers, MA), β-actin (1:1000; Santa Cruz Biotechnology), anti-MCL1 (1:1000; Cell Signaling), and anti-BCL2 (1:1000; Cell Signaling). After washing, the membrane was incubated with a species-specific horseradish peroxidase-conjugated secondary antibody (1:3000; Cell Signaling Technology, Danvers, MA, USA) for 1 h at room temperature. The membrane three times with TBS-T, and ECL substrate (Amersham Cytiva, USA) was added. Membrane scans were performed using the ChemiDoc imaging system (Cytiva, USA). The Western blot experiments were performed at least three times to measure expression levels. Western blot data were quantified using the ImageJ software (National Institutes of Health, Bethesda, MD, USA). The uncropped western blots from the different figures are shown in Supplementary Fig. 8.

### Flow cytometry for analyzing apoptosis

HNSCC cells were plated in 6-well plates and cultured to 70–80% confluence. Cells were transfected with siGFP- or FLOT1-specific siRNAs and maintained for 24 h. The cells were irradiated with 4 Gy using 250-kVp X-rays. Forty-eight hours after irradiation, the cells were harvested, and apoptosis was detected using the Annexin V-FITC Apoptosis Detection Kit (BioBud, Seongnam, Korea), according to the manufacturer’s recommendations.

### Preparation of chitosan hydrogel loaded with siRNA

A chitosan hydrogel (CH), which was previously reported to have stable physical characteristics as an in vitro or in vivo depot system after intratumoral injection, was used [61]. The CH solution had the following properties: medium molecular weight of 161 kDa, viscosity of 200,000 cps, and degree of deacetylation of 80%. To prepare this solution, CH (Sigma–Aldrich) was dissolved in 1% acetic acid. Next, a solution of tripolyphosphate (TPP, Sigma-Aldrich, Burlington, MA, USA) containing siRNA was prepared by dissolving 0.2 g of TPP in 0.2 ml of distilled water. The CH solution was cooled to 4 °C and continuously stirred while adding 0.2 ml of TPP. CH was successfully formed in vivo at body temperature and physiological pH following intratumoral injection into tumor-bearing mice.

### Xenograft mouse model

The study utilized female BALB/c nude mice weighing 20 ± 2 g, procured from JunbioTech (Daego, Korea), and conducted in accordance with the policies outlined by the Kyung Hee Medical Center Institutional Animal Care and Use Committee (KHMC-IACUC-23-003). Eight mice were randomly allocated to each group, with two mice per group. CAL27.RR cells were subcutaneously injected into the right and left thighs of each mouse at a dose of 2.0 × 10^6^ cells per mouse in 0.1 ml of saline. Subsequently, tumors were allowed to develop. After 3 days, the mice received treatment via intratumoral injection of CH with siGFP and CH with siFLOT1 at a dose of 50 µl/ml. Additionally, the tumors were exposed to ionizing radiation (2 Gy daily for 5 days, for a total of 10 Gy). A 5 × 2 Gy-fractionated IR dose was used to replicate the treatment protocol typically administered to patients for over a week. The weights of the animals were recorded twice a week. The longest and shortest tumor lengths (long and short) were measured twice a week at right angles using electronic calipers and converted to volume using the following formula: volume = [(short)^2^ × (long)]/2.

### Statistical analysis

The LASSO regression model was implemented using the glmnet package (version 4.0–2, https://cran.r-project.org/web/packages/glmnet/index.html), and the gene signature was derived using 10-fold cross-validation. The Kaplan–Meier method was employed to generate OS and recurrence-free survival (RFS) curves for each subgroup in each cohort. The log-rank test was used to compare the OS and RFS rates among the subgroups. Univariate and multivariate Cox proportional hazards models were used to assess independent prognostic factors associated with the survival of HNSCC patients. Results from the Cox regression analyses are presented as hazard ratios (HRs), 95% confidence intervals (95% CIs), and *p* values. Statistical analyses were performed using R software (http://www.r-project.org). All in vitro experiments were conducted at least three times. Differences between groups were assessed using a two-sided Student’s *t*-test for comparisons involving two groups and ANOVA for comparisons involving three groups. Statistical significance was set at *P* < 0.05. significant. Statistical analyses and chart creation were performed using R Statistics (version 4.2.1) and Prism 10 (GraphPad, CA, USA).

## Supplementary information

Supplementary information

Original data

## Data Availability

The data that support the findings of this study are openly available in the following databases: TCGA database (https://xenabrowser.net/), Greece database, FHCRC database, and UNC database (https://www.ncbi.nlm.nih.gov/gds), and CCLE database (https://portals.broadinstitute.org/ccle).
