# Supplementary material for: Prognostic value of FLOT1-related gene signature in head and neck squamous cell carcinoma: insights into radioresistance mechanisms and clinical outcomes
Source: Cell Death Discov. 2025 May 7;11:224. doi: 10.1038/s41420-025-02500-1 (PMC12058980; doi:10.1038/s41420-025-02500-1)

**Prognostic Value of FLOT1-Related Gene Signature in Head and Neck Squamous Cell Carcinoma: Insights into Radioresistance Mechanisms and Clinical Outcomes**

**Supplementary Information**

**Supplementary materials and methods**

**Cell culture and transfection**

The SNU1041 cell line was provided by Prof. Chul Ho Kim (Aju University). The SNU1076 cell line was purchased from the Korean Cell Line Bank (KCLB), while SCC4 and CAL27 cell lines were purchased from the American Type Culture Collection (ATCC). SNU1041 and SNU1076 cells were cultured in the RPMI medium. (Corning, Manassas, VA, USA) SCC4 cells were cultured in DMEM/F12 medium (Corning). CAL27 cells were cultured in DMEM/HIGH GLUCOSE medium (Corning). All cells were supplemented with 10% Fetal Bovine Serum (FBS, Corning) and 1% penicillin-streptomycin (PS, Corning), and maintained in media specific to each cell line at 37°C in a humidified atmosphere containing 5% CO_2_. All cell lines were purchased within the last 5 years, and their identities were confirmed by short tandem repeat (STR) profiling using KCLB. The siRNAs specific for FLOT1 (5′-rCrCrC rUrCrA rArUrG rUrCrA rArGrA rGrUrG rArArA rArGG T-3′) were designed and synthesized by IDT (Cambridge, MA, USA). siRNAs specific to GFP (5′-GCAUCAAGGUGAACUUCAA-3′) were obtained from Bioneer (Daejeon, Korea). Transfection of control-siGFP and FLOT1-siRNA was performed using Lipofectamine RNAiMax (Invitrogen), following the manufacturer’s instructions. Knockdown of FLOT1 was further confirmed by western blotting.

**MTT Assay**

To evaluate the effect of bpvPICs on cell viability, MTT assays were conducted using an EZ-Cytox Cell Viability Assay Kit (Daeli Lab Service, Seoul, Republic of Korea). HNSCC cells were seeded at a density of 5,000 cells/well in 96-well plates and treated with bpvPICs (1.25, 2.5, 5, 10, 20, and 40 nM/ml). Following a 24-hour incubation period in a humidified atmosphere containing 5% CO_2_ at 37°C, cell viability was assessed using the MTT assay, with absorbance measured at 570 nm. bpvPIC was purchased from Merck (Darmstadt, Germany).

**Colony formation assay**

A colony forming assay (CFA) was performed to assess the ability of cells to form colonies. GFP or FLOT1-specific siRNA-treated cells were plated in 6-well plates at a density of 300 cells per well in growth medium supplemented with 10% FBS and 1% PS, then incubated at 37°C. After 24 h, the cells were irradiated with 0, 2, 4, or 8 Gy radiation. Following irradiation, all cells were maintained at 37°C for 10–14 d. The cells were then stained with crystal violet for 10 min at room temperature. All experiments were conducted in triplicate. The significance of the differences between dose responses was determined using two-way ANOVA.

**Western blotting**

HNSCC cells were lysed in RIPA buffer (50 mM Tris/HCl, 150 mM NaCl, 2 mM EDTA, and 1% Triton™ X-100) supplemented with protease inhibitors (Roche, Mannheim, Germany) and phosphatase inhibitors (Sigma-Aldrich, Burlington, MA, USA). Protein concentrations were determined using the BCA protein assay kit (Thermo Fisher Scientific, Waltham, MA, USA). For western blotting, 15 μg of protein was mixed with SDS sample buffer (Invitrogen), boiled for 10 minutes at 100°C, and electrophoresed on an 8–15% gradient bis-Tris gel. Subsequently, the proteins were transferred onto polyvinylidene fluoride membranes (Millipore, Billerica, MA, USA). After blocking the membrane with Tris-buffered saline (TBS) containing 5% nonfat dry milk, it was incubated overnight at 4°C with the following primary antibodies: anti-FLOT1 (1:1000; Cell Signaling), anti-PTEN (1:1000; Santa Cruz Biotechnology, Santa Cruz, CA, USA), anti-IGF1R (1:1000; Santa Cruz Biotechnology), anti-pPTEN (1:1000; Cell Signaling Technology, Danvers, MA), β-actin (1:1000; Santa Cruz Biotechnology), anti-MCL1 (1:1000; Cell Signaling), and anti-BCL2 (1:1000; Cell Signaling). After washing, the membrane was incubated with a species-specific horseradish peroxidase-conjugated secondary antibody (1:3000; Cell Signaling Technology, Danvers, MA, USA) for 1 h at room temperature. the membrane 3 times with TBS-T, and ECL substrate (Amersham Cytiva, USA) was added. Membrane scans were performed using the ChemiDoc imaging system (Cytiva, USA). Western blot data were quantified using the ImageJ software (National Institutes of Health, Bethesda, MD, USA).

**Flow cytometry for analyzing apoptosis**

HNSCC cells were plated in 6-well plates and cultured to 70–80% confluence. Cells were transfected with siGFP- or FLOT1-specific siRNAs and maintained for 24 h. The cells were irradiated with 4 Gy using 250-kVp X-rays. 48 hours after irradiation, the cells were harvested, and apoptosis was detected using the Annexin V-FITC Apoptosis Detection Kit (BioBud, Seongnam, Korea), according to the manufacturer’s recommendations.

**Preparation of Chitosan hydrogel loaded with siRNA**

A chitosan hydrogel (CH), which was previously reported to have stable physical characteristics as an in vitro or in vivo depot system after intratumoral injection, was used. (19) The CH solution had the following properties: medium molecular weight of 161 kDa, viscosity of 200,000 cps, and degree of deacetylation of 80%. To prepare this solution, CH (Sigma–Aldrich) was dissolved in 1% acetic acid. Next, a solution of tripolyphosphate (TPP, Sigma-Aldrich, Burlington, MA, USA) containing siRNA was prepared by dissolving 0.2 g of TPP in 0.2 ml of distilled water. The CH solution was cooled to 4°C and continuously stirred while adding 0.2 ml of TPP. CH was successfully formed in vivo at body temperature and physiological pH following intratumoral injection into tumor-bearing mice.

**Xenograft mouse model**

The study utilized female BALB/c nude mice weighing 20 ± 2 g, procured from JunbioTech (Daego, Korea), and conducted in accordance with the policies outlined by the Kyung Hee Medical Center Institutional Animal Care and Use Committee (KHMC-IACUC-23-003). To comply with these policies, we used the minimum number of animals necessary and performed statistical analysis. Consequently, we determined the minimum animal sample size for the analysis and selected eight mice. Eight mice were randomly allocated to each group with two mice per group. CAL27.RR cells were subcutaneously injected into the right and left thighs of each mouse at a dose of 2.0 × 10^6^ cells per mouse in 0.1 ml of saline. Subsequently, tumors were allowed to develop. After 3 days, the mice received treatment via intratumoral injection of CH with siGFP and CH with siFLOT1 at a dose of 50 µl/ml. Additionally, the tumors were exposed to ionizing radiation (2 Gy daily for 5 days, for a total of 10 Gy). A 5× 2 Gy-fractionated IR dose was used to replicate the treatment protocol typically administered to patients for over a week. The weights of the animals were recorded twice a week. The longest and shortest tumor lengths (long and short) were measured twice a week at right angles using electronic calipers and converted to volume using the following formula: volume = [(short)^2^ × (long)]/2. The measurement of tumor volumes in the mice was performed by a someone blinded.

**Supplementary Figure legends**

**Supplementary Figure 1.**

Kaplan–Meier survival curves for FLOT1 expression. The significance was calculated using the log-rank test.

**Supplementary Figure 2.**

Association of FLOT1-related gene signature with clinical stage of HNSCC patients. Log-rank test was used to estimate the P value. (A) The FLOT1-HR subgroup showed significantly lower 5-years OS rates than the FLOT1-LR subgroup in HNSCC patients with an advanced clinical stage disease (P = 0.0093). (B) In early-stage HNSCC patients, there were no significant differences in f5-years OS rates between the FLOT1-HR and FLOT1 LR subgroup. (P = 0.73)

**Supplementary Figure 3.**

Validation of the FLOT1-Related Gene Signature in RT and chemoradiotherapy

(A, B) Comparison of 5-year OS and RFS rates between the FLOT1-HR and FLOT1-LR subgroups in HNSCC patients treated with chemoradiotherapy from the TCGA cohort (n = 60, p < 0.05).

(C, D) Comparison of 5-year OS and RFS rates between the FLOT1-HR and FLOT1-LR subgroups in HNSCC patients treated with RT-only from the TCGA cohort (n = 185, p < 0.05). Significance was calculated using the log-rank test.

**Supplementary Figure 4.**

The response to RT between HPV-positive and HPV-negative HNSCC patients

The 5-year overall survival (OS) rates for the FLOT1-HR subgroup and FLOT1-LR subgroup in HPV-positive patients in TCGA (n = 69, p < 0.05). (B) The 5-year OS rates for the FLOT1-HR subgroup and FLOT1-LR subgroup in HPV-negative patients in TCGA (n = 274, p < 0.05). (C) Kaplan-Meier plot of the FLOT1-HR subgroup and FLOT1-LR subgroup of HPV-positive patients receiving RT in TCGA (n = 42, p < 0.05). (D) Kaplan-Meier plot of the FLOT1-HR subgroup and FLOT1-LR subgroup of HPV-positive patients not receiving RT in TCGA (n = 15, p > 0.05). (E) Kaplan-Meier plot of the FLOT1-HR subgroup and FLOT1-LR subgroup of HPV-negative patients receiving RT in TCGA (n = 134, p < 0.05). (F) Kaplan-Meier plot of the FLOT1-HR subgroup and FLOT1-LR subgroup of HPV-negative patients not receiving RT in TCGA (n = 90, p > 0.05). Significance was calculated using the log-rank test.

**Supplementary Figure 5.**

Evaluation of FLOT1 levels in HNSCC.

(A) Classification of 15 HNSCC cell lines into FLOT1 HR and LR subgroups. The cell lines were divided into FLOT1 HR and LR subgroups based on the BCCP algorithm.

(B) Comparison of FLOT1 expression levels among the 15 HNSCC cell lines. The proteins were analyzed using western blotting. β-Actin was included as an internal loading control. Protein levels were measured using ImageJ.

**Supplementary Figure 6.**

MTT assay results demonstrating the impact of bpvPIC on cell viability in HNSCC cells. Cells were treated with various concentrations of bpvPIC A for 24 hours. Cell viability was assessed using an ELISA plate reader at 570 nm. Values represent the mean ± SD of three independent experiments.

**Supplementary Figure 7.**

Cells were transfected with siGFP (100 nM) as a negative control and siFLOT1 (100 nM). Twenty-four hours after FLOT1 expression was inhibited, the cells were treated with the PTEN activation inhibitor bpvPIC. Then, 4Gy was irradiated in CAL27 cells. The results in the graphs represent three independent experiments performed in triplicate. P < 0.05, **P < 0.01, and ***P < 0.001, using a 2-tailed Student’s t test.

**Supplementary Figure 8.**

The Hedges' g coefficients were calculated by multiplying Cohen's d by a correction factor, as described by Borenstein et al. (2009). Additionally, variance, standard error, and 95% confidence intervals for Hedges' g were calculated. Hedges' g can be interpreted as small (under 0.2), medium (under 0.5), and large (under 0.8) (Ellis, 2010).

**Supple figures**

**Supplementary Figure 1.**


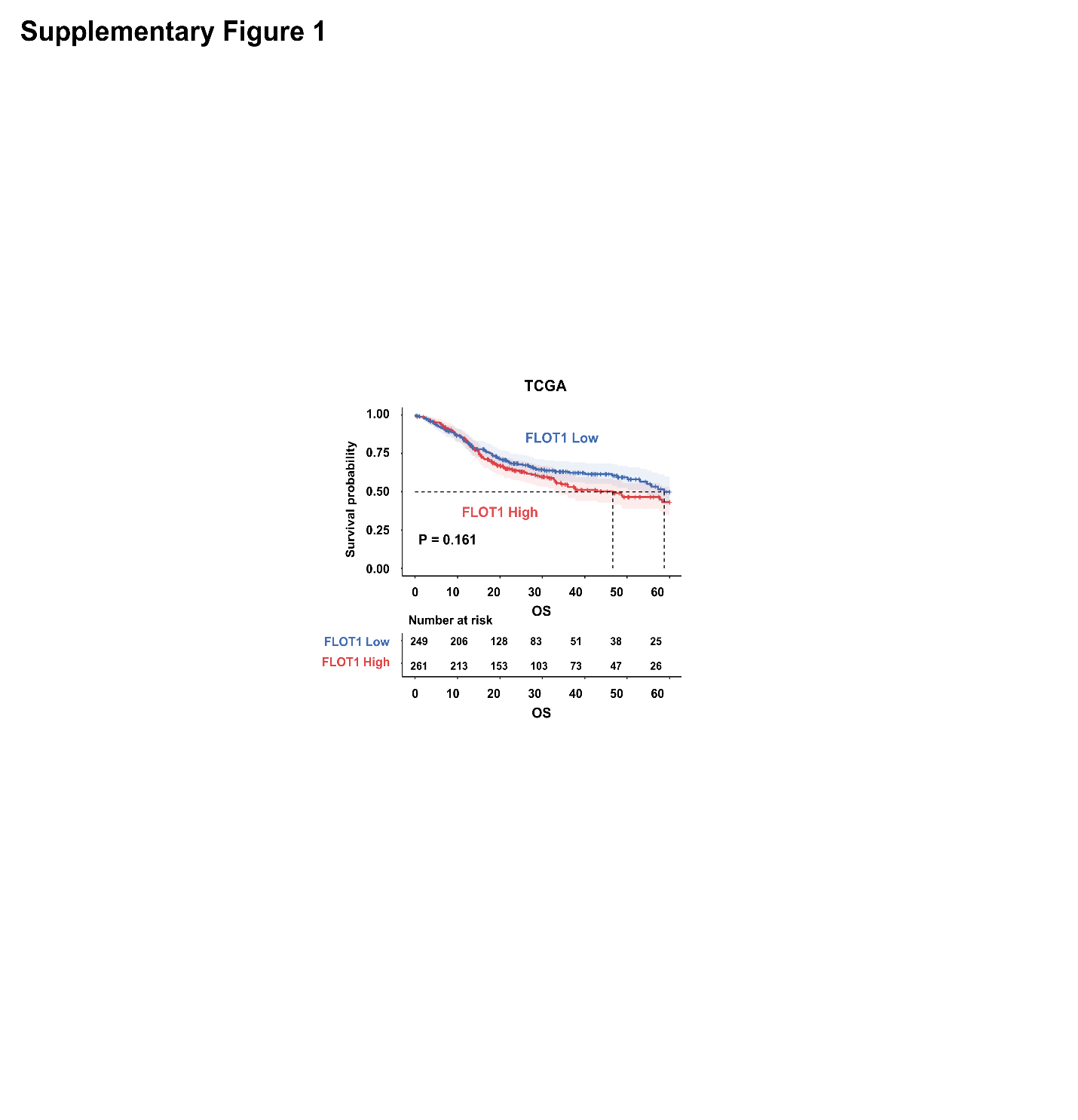


**Supplementary Figure 2.**


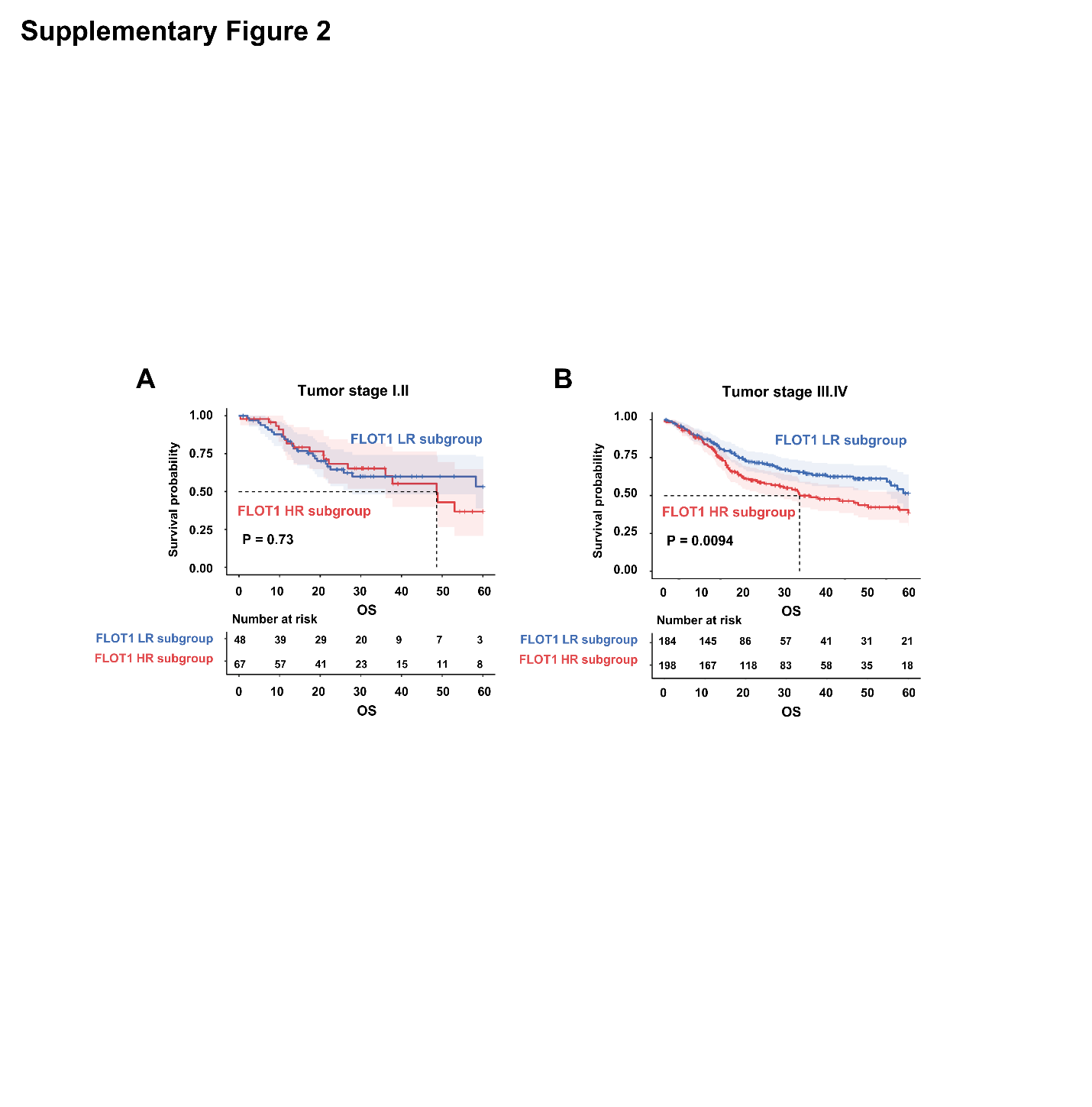


**Supplementary Figure 3.**

**
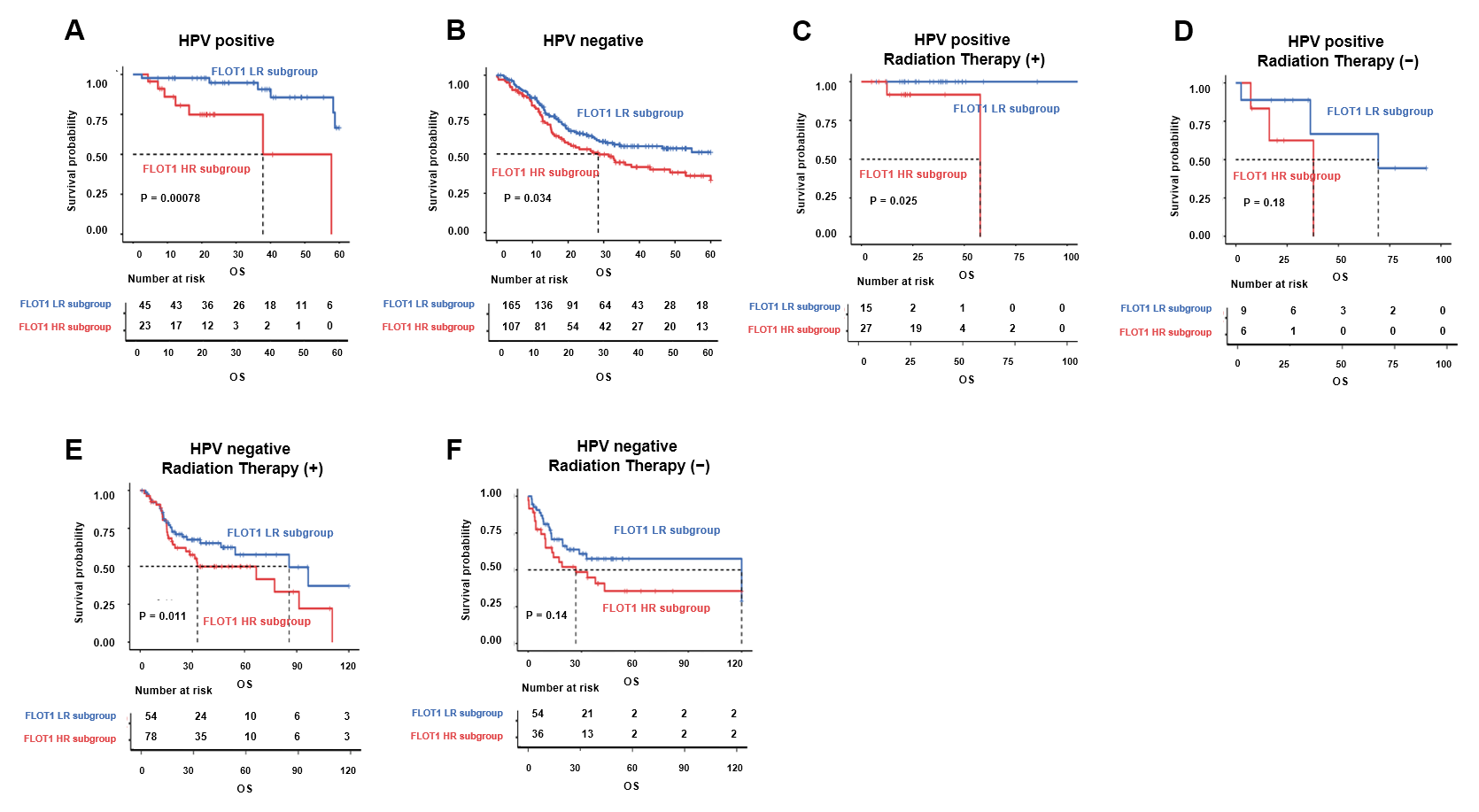
**

**Supplementary Figure 4.**

**
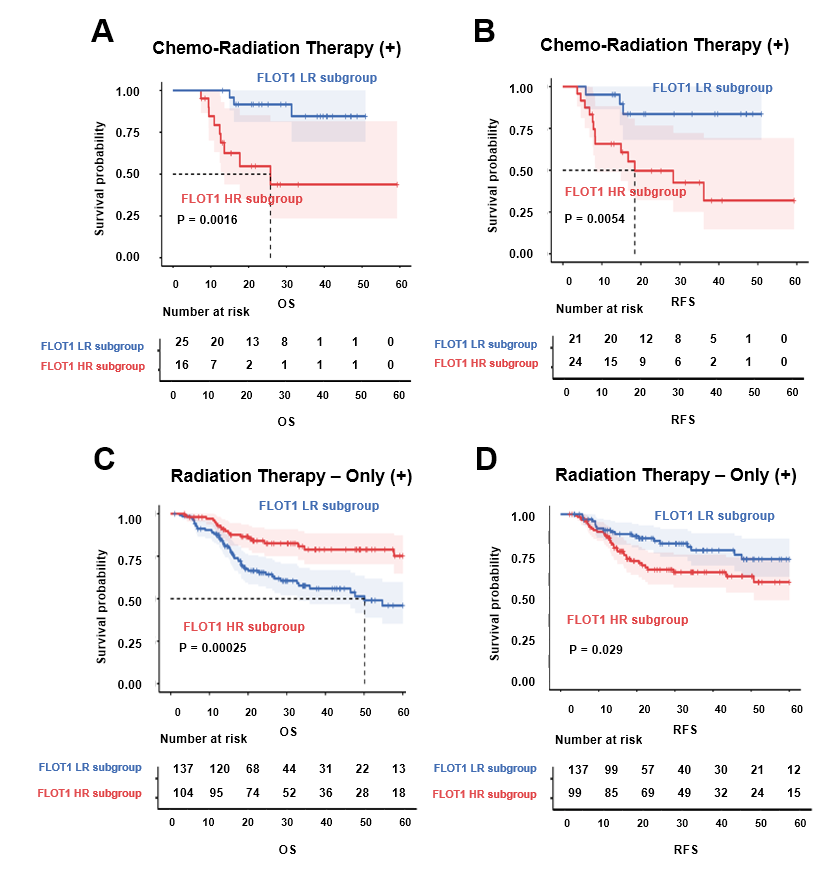
**

**Supplementary Figure 5.**


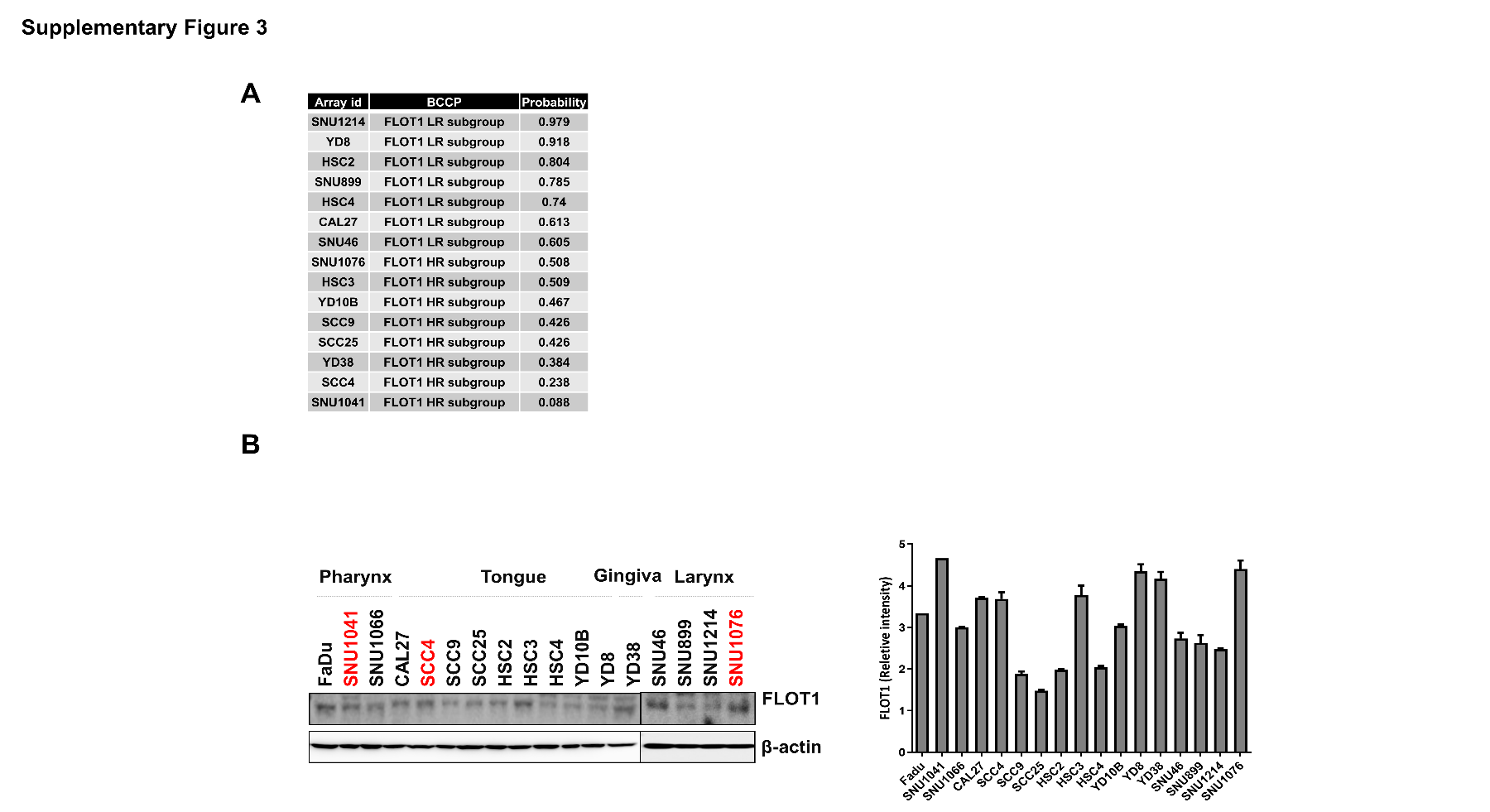


**Supplementary Figure 6.**


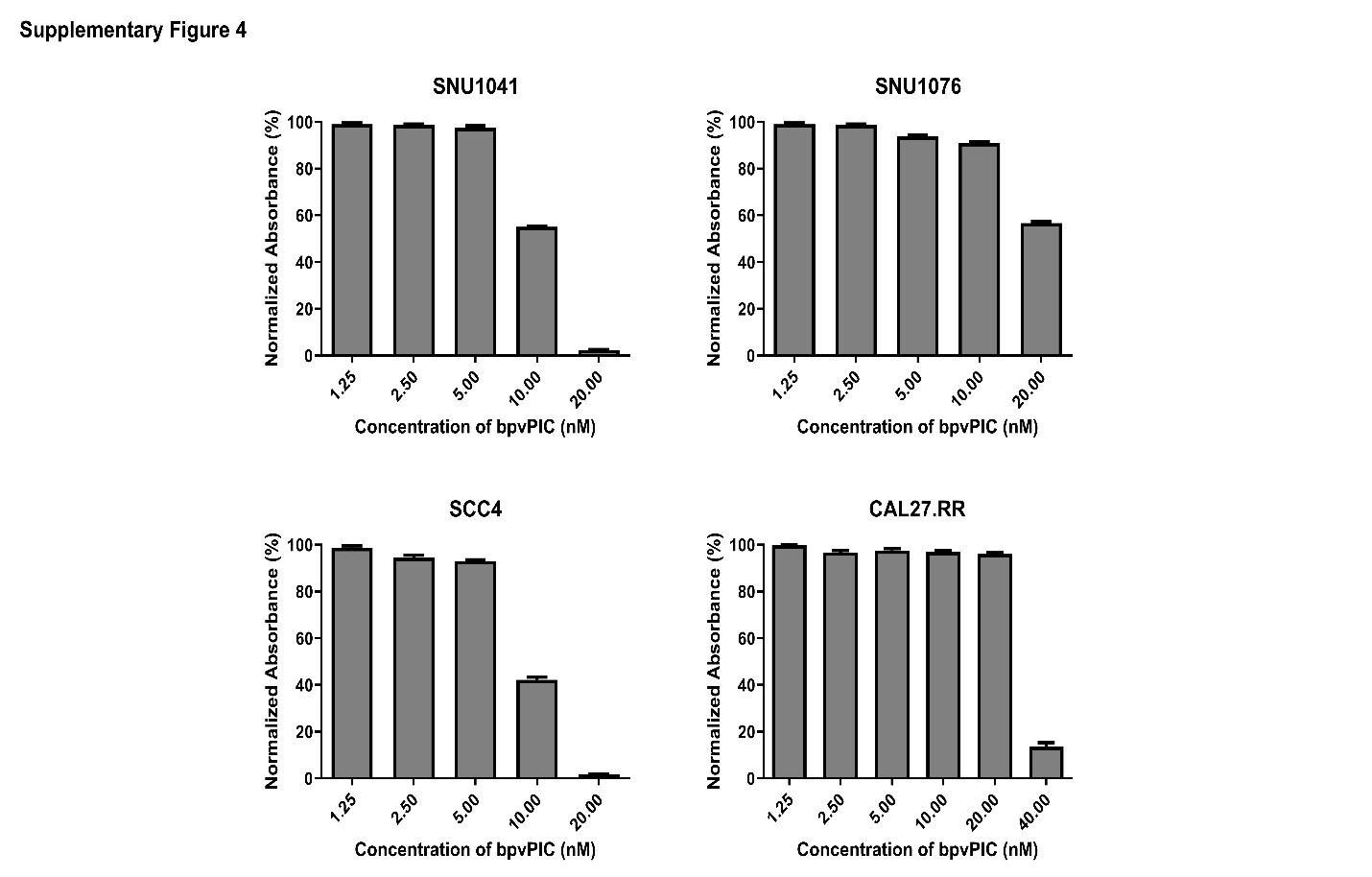


**Supplementary Figure7**

**
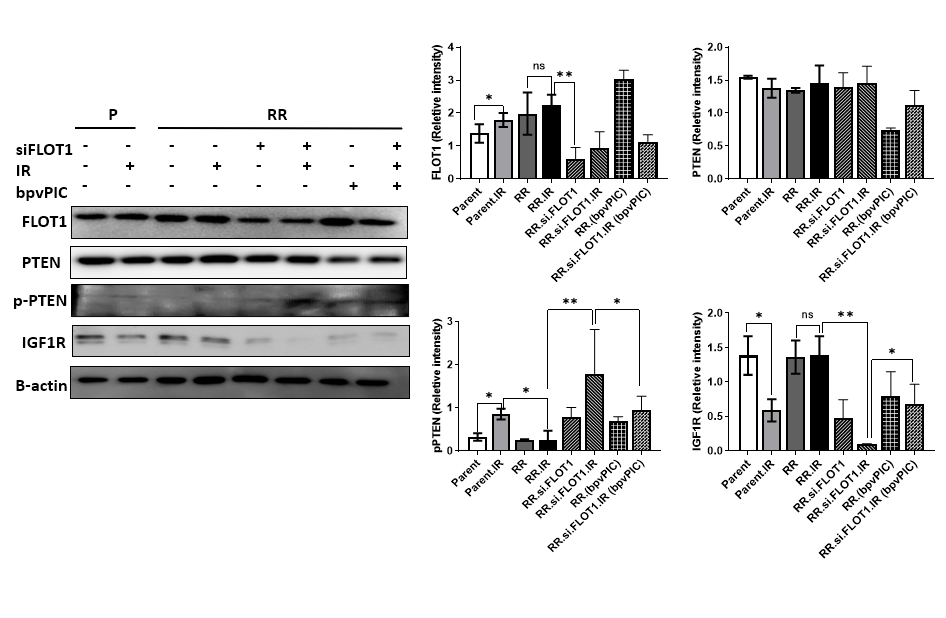
**

**Supplementary Figure 8**


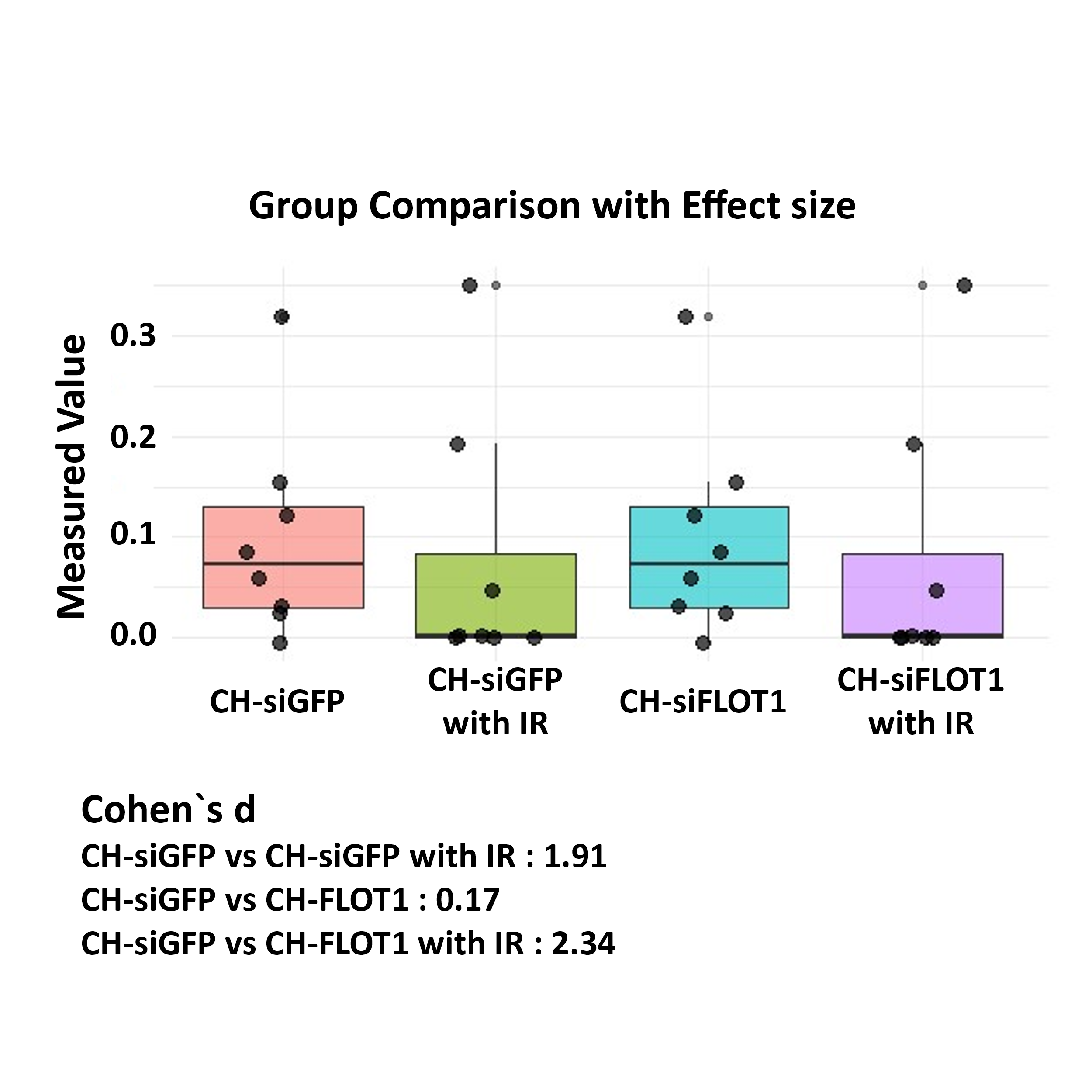

Supplement: Supplementary file 1 — Supplementary information [file 41420_2025_2500_MOESM1_ESM.docx]
