# Supplementary material for: Prognostic value of FLOT1-related gene signature in head and neck squamous cell carcinoma: insights into radioresistance mechanisms and clinical outcomes
Source: Cell Death Discov. 2025 May 7;11:224. doi: 10.1038/s41420-025-02500-1 (PMC12058980; doi:10.1038/s41420-025-02500-1)
Supplement: Supplementary file 2 — Original data [file 41420_2025_2500_MOESM2_ESM.docx]

**Figure 5A**

**
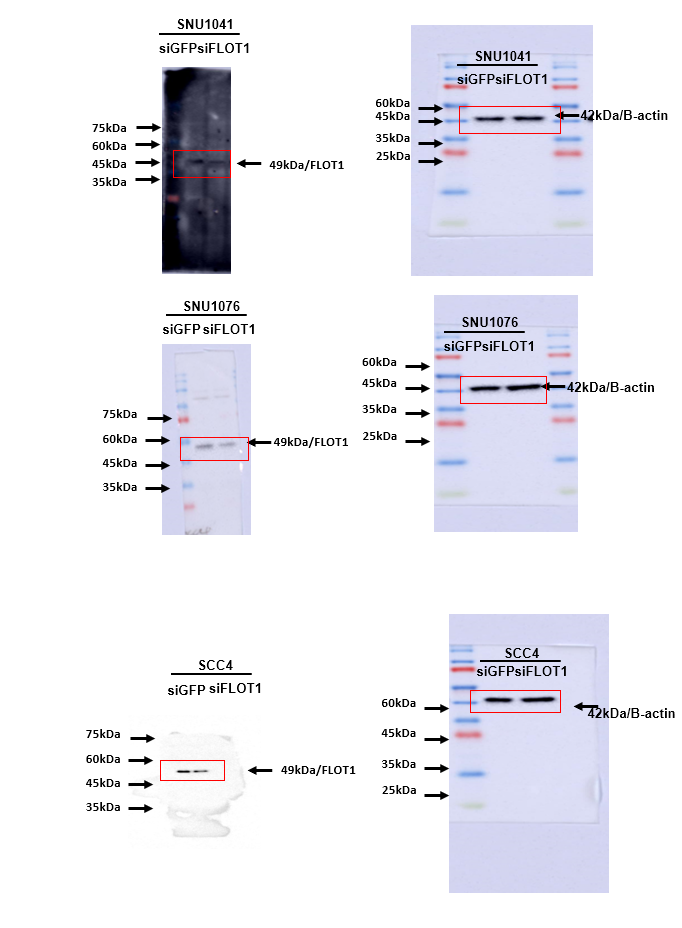
**

**Figure 5C**

**
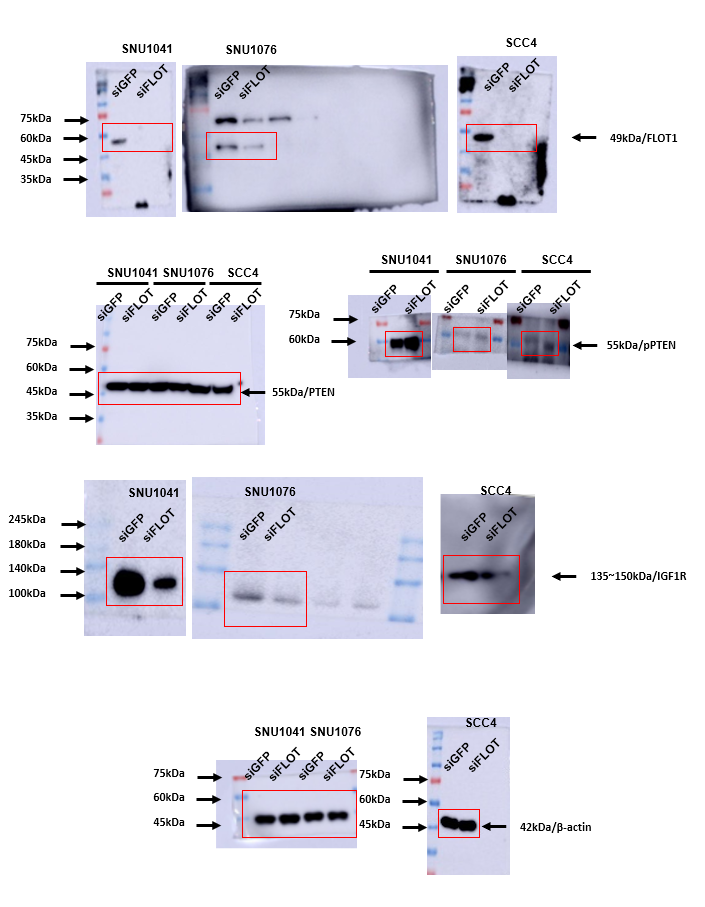
**

**Figure 5D**

**
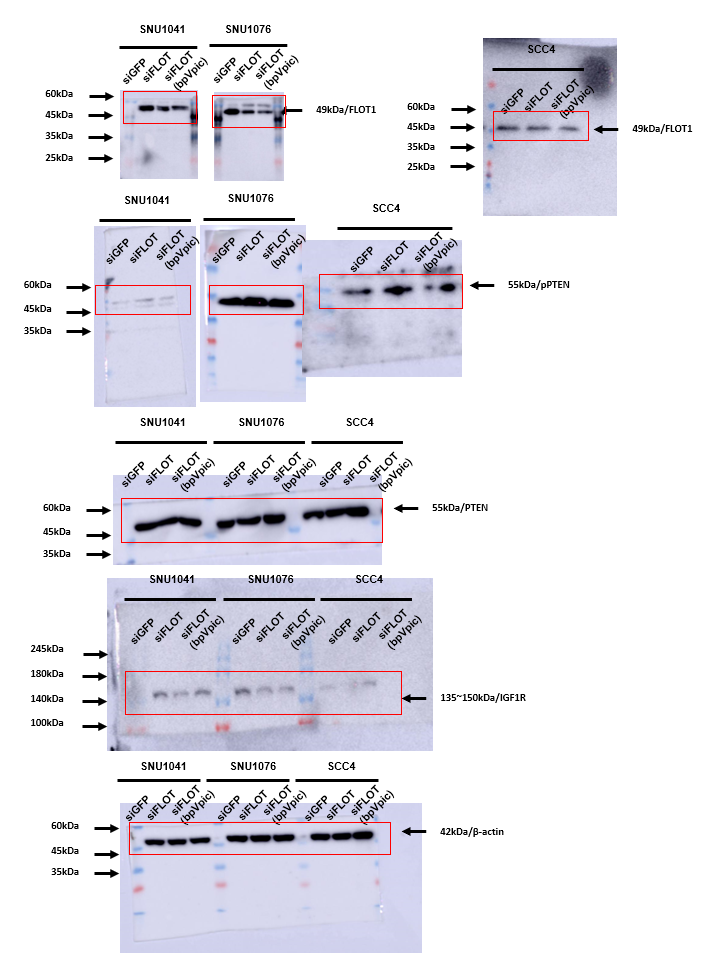
**

**Figure 6B**

**
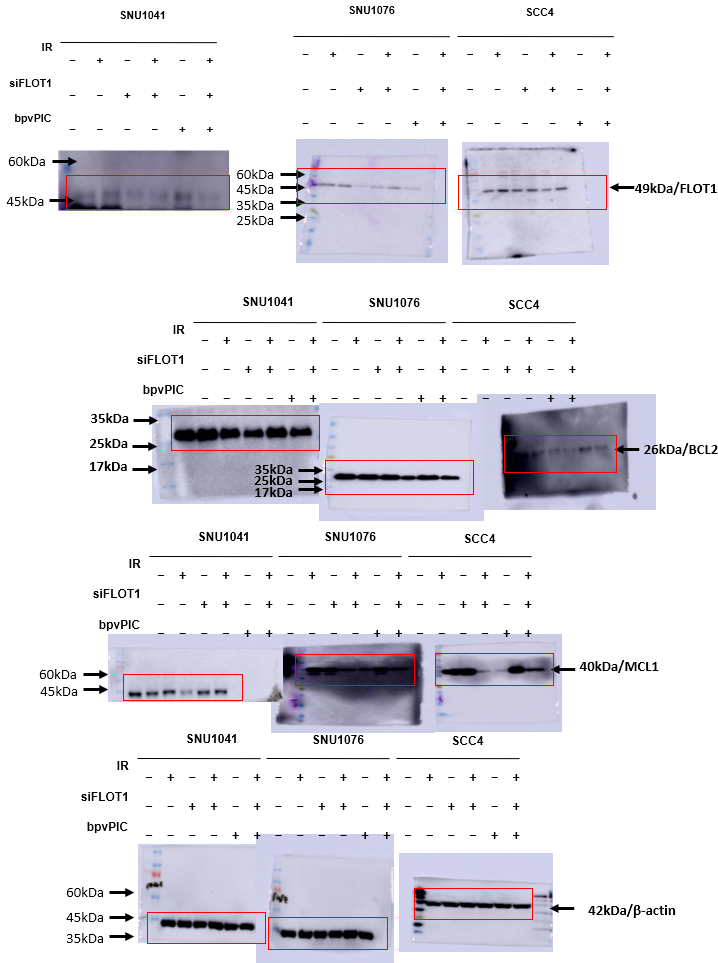
**

**Figure 7A**

**
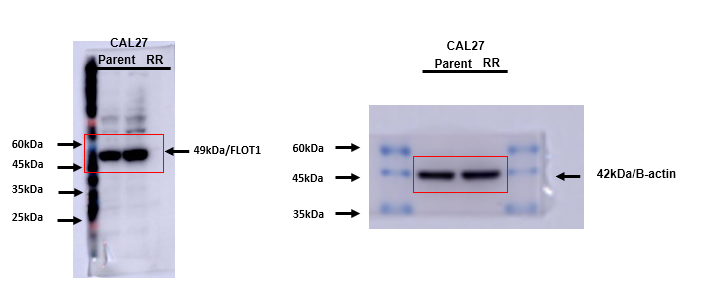
**

**Figure 7C**

**
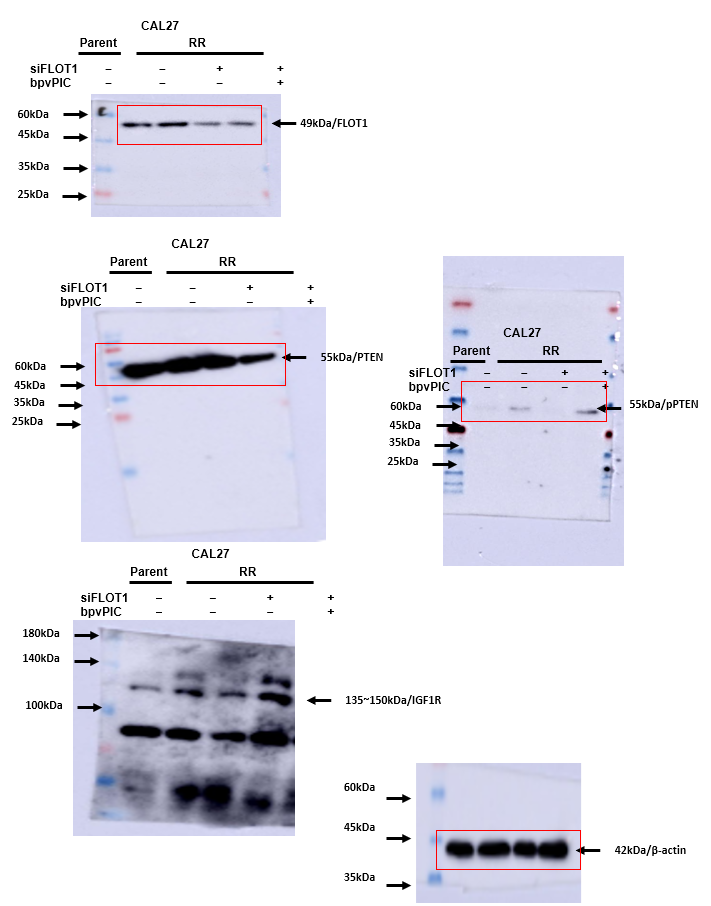
**

**Figure 7E**

**
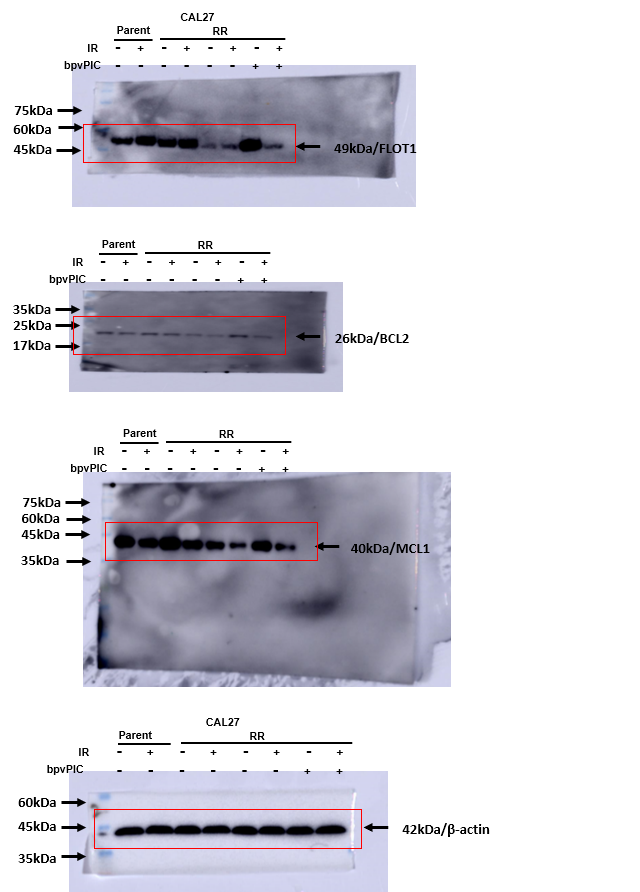
**

**Supplementary Figure 6B**

**
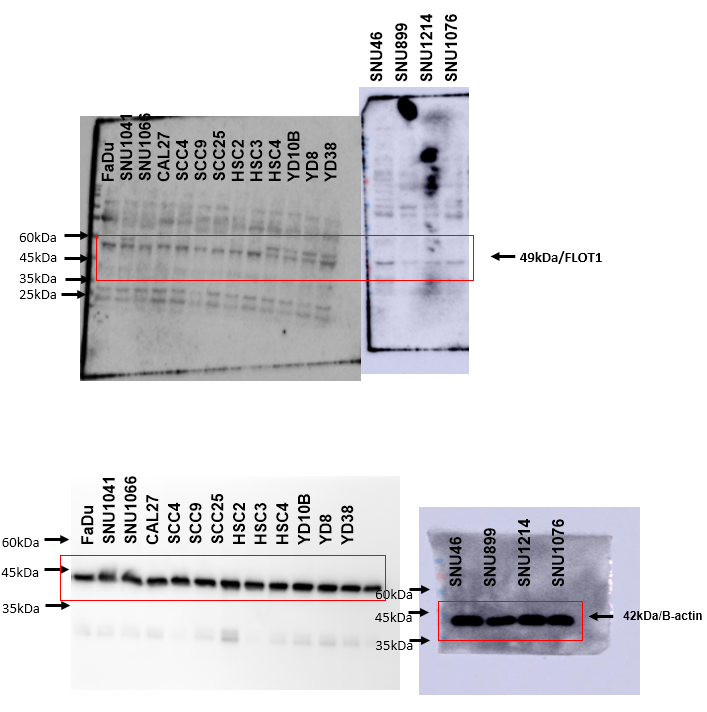
**

**Supplementary Figure 7**

**
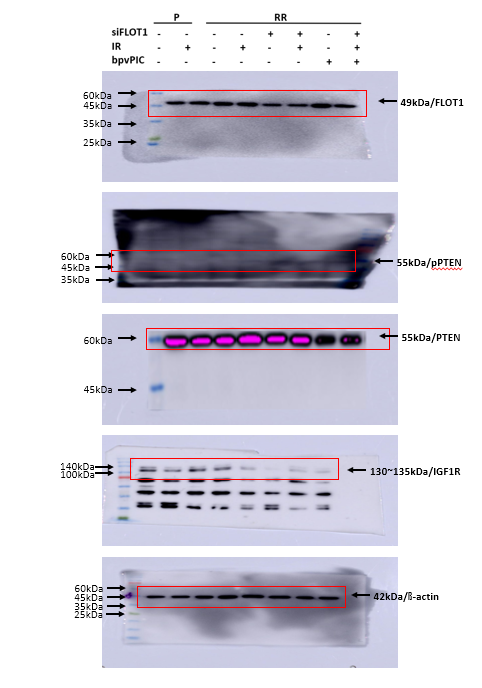
**
